# Tumor size measurements of pancreatic cancer with neoadjuvant therapy based on RECIST guidelines: is MRI as effective as CT?

**DOI:** 10.1186/s40644-023-00528-z

**Published:** 2023-01-18

**Authors:** Panpan Yang, Kuanzheng Mao, Yisha Gao, Zhen Wang, Jun Wang, Yufei Chen, Chao Ma, Yun Bian, Chengwei Shao, Jianping Lu

**Affiliations:** 1 Department of Radiology, Changhai Hospital of Shanghai, Naval Medical University, No. 168 Changhai Road, Shanghai, 200433 China; 2 School of Health Science and Engineering, University of Shanghai for Science and Technology, Shanghai, China; 3 Department of Pathology, Changhai Hospital of Shanghai, Naval Medical University, Shanghai, China; 4 College of Electronic and Information Engineering, Tongji University, Shanghai, China

**Keywords:** Pancreatic cancer, Neoadjuvant therapy, CT, MRI, Tumor size

## Abstract

**Objectives:**

To compare tumor size measurements using CT and MRI in pancreatic cancer (PC) patients with neoadjuvant therapy (NAT).

**Methods:**

This study included 125 histologically confirmed PC patients who underwent NAT. The tumor sizes from CT and MRI before and after NAT were compared by using Bland–Altman analyses and intraclass correlation coefficients (ICCs). Variations in tumor size estimates between MRI and CT in relationship to different factors, including NAT methods (chemotherapy, chemoradiotherapy), tumor locations (head/neck, body/tail), tumor regression grade (TRG) levels (0–2, 3), N stages (N0, N1/N2) and tumor resection margin status (R0, R1), were further analysed. The McNemar test was used to compare the efficacy of NAT evaluations based on the CT and MRI measurements according to RECIST 1.1 criteria.

**Results:**

There was no significant difference between the median tumor sizes from CT and MRI before and after NAT (*P* = 0.44 and 0.39, respectively). There was excellent agreement in tumor size between MRI and CT, with mean size differences and limits of agreement (LOAs) of 1.5 [-9.6 to 12.7] mm and 0.9 [-12.6 to 14.5] mm before NAT (ICC, 0.93) and after NAT (ICC, 0.91), respectively. For all the investigated factors, there was good or excellent correlation (ICC, 0.76 to 0.95) for tumor sizes between CT and MRI. There was no significant difference in the efficacy evaluation of NAT between CT and MRI measurements (*P* = 1.0).

**Conclusion:**

MRI and CT have similar performance in assessing PC tumor size before and after NAT.

**Supplementary Information:**

The online version contains supplementary material available at 10.1186/s40644-023-00528-z.

## Introduction

As a lethal cancer with poor prognosis and high mortality, pancreatic cancer (PC) results in more than 460,000 deaths a year worldwide [1] and may be the second leading cause of death due to cancer by 2030 in the United States [2]. According to the cancer statistics of the United States in 2022, the 5-year survival rate of PC is only 11% [3]. Despite surgical resection is the possible cure method for PC, nowadays the therapeutic principle clearly is not always operation first, considering neoadjuvant therapy (NAT). Most patients with PC have local tumor progression or distant metastasis at diagnosis and miss the opportunity for surgical resection. Only approximately 10%-15% of the patients who are evaluated as having resectable PC can undergo radical surgical resection. More patients are diagnosed with borderline resectable or locally advanced PC [4]. To improve the management of this lethal tumor, on the one hand, earlier diagnosis of resectable PC should be explored, while, on the other hand, new treatment methods should be developed.

NAT is a treatment strategy for resectable, borderline resectable and locally advanced PC that has been emerging considerably [5–9]. The definition of NAT for PC is preoperative chemotherapy with or without radiation therapy [10]. The purpose of NAT for PC is to reduce the tumor stage, improve the rate of R0 resection (a microscopically margin-negative resection), and reduce postoperative recurrence and metastasis [11–14]. Ideal methods for reassessment after NAT for PC are lacking, and currently, the response evaluation criteria in solid tumors (RECIST) are still the main choice for use [9, 15]. Investigators could adopt RECIST criteria to assess the treatment outcomes including complete response (CR), partial response (PR), stable disease (SD) and progressive disease (PD) after NAT for PC patients with tumor size measurements. CT and MRI are the commonly used imaging modalities for the diagnosis and evaluation of PC and show similar effects in the staging and diagnosis of pancreatic cancer, both pre-NAT and preoperation without NAT [16]. Different from other solid tumors, the changes in tissue components of PC after NAT, including tumor necrosis, oedema, inflammation, and fibrosis, result in significant effects on CT-image evaluations of tumor treatment and resectability [17]. The accurate staging of tumors with CT or MRI is used not only for the selection of treatment methods but also for accurate tumor measurement to improve outcomes in PC patients after NAT in clinical practice. In this study, we investigated the similarities and differences in tumor measurements between CT and MRI before and after NAT for PC based on RECIST 1.1 criteria.

## Methods

### Patients

**Fig. 1:**
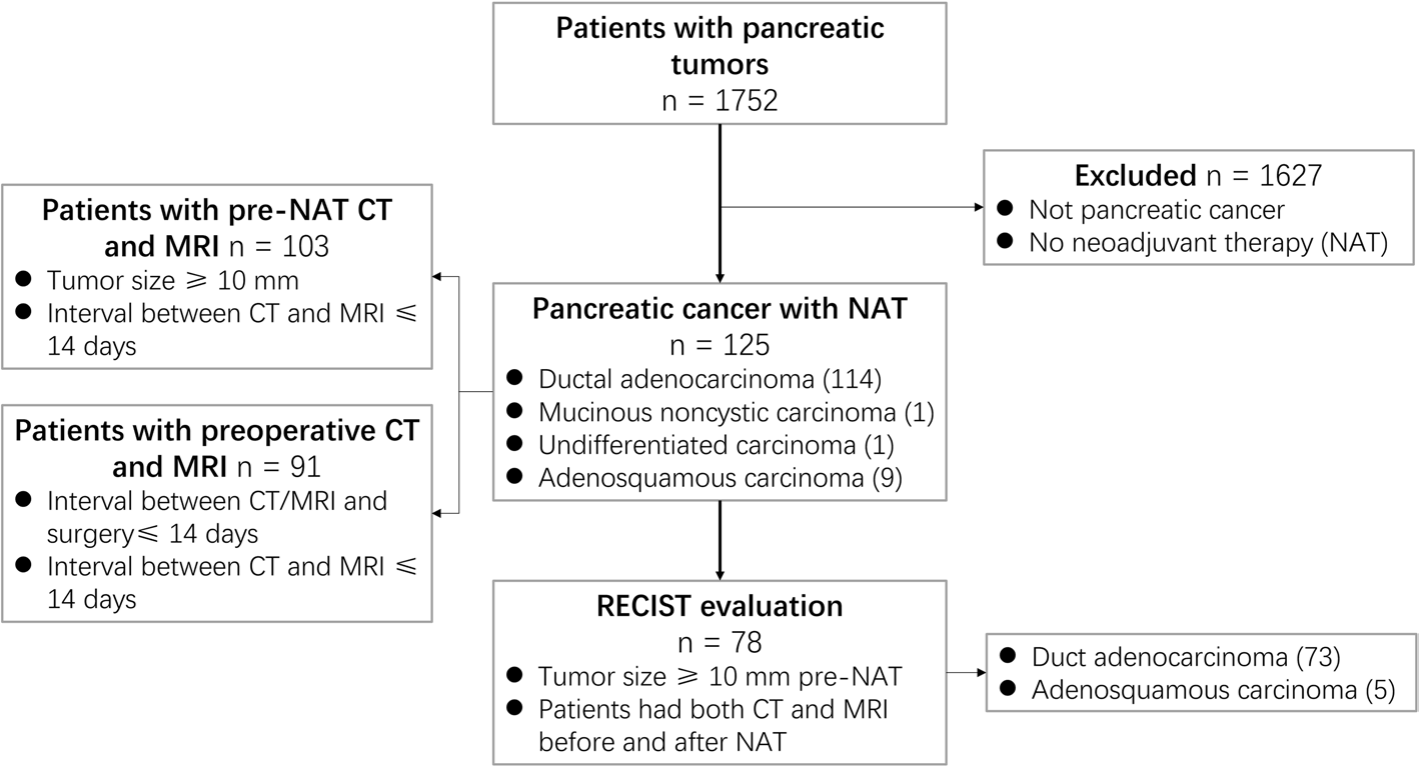
Flow chart of the patient selection process

Our institutional review board approved this retrospective study. We undertook a review of all patients who had undergone pancreatic contrast-enhanced MRI or contrast-enhanced CT and pancreatic tumor resection between April 2019 and December 2021 and identified 1752 pancreatic tumor patients. Among these patients, 125 subjects (72 males, 53 females; mean age: 60.6 ± 8.5 years; range: 33–76 years) had received NAT and had detailed pathological reports. Before NAT, 104 patients underwent both contrast-enhanced CT and contrast-enhanced MRI examinations within an interval of less than 14 days. After NAT, a total of 109 patients underwent both preoperative contrast-enhanced CT and contrast-enhanced MRI examinations, and 17 patients were excluded due to an MRI or CT examination time more than 14 days before the day of the operation. Additionally, a patient with tumor sizes less than 10 mm per pre-NAT CT and MRI measurements was excluded. Finally, this study included 103 and 91 patients who had received both CT and MRI examinations in the pre-NAT group and the finished-NAT group, respectively. Among the 125 enrolled cases, a total of 78 patients who underwent both CT and MRI within an interval of less than 14 days before NAT and subsequently underwent preoperative CT and MRI less than 14 days before the day of the operation were chosen for further analyses (Fig. 1, Table 1).

**Table 1:** Characteristics of 125 patients with pancreatic cancer

Mean age ± SD, years (range)	60.6 ± 8.5 (33–76)
Gender, n (%)	
Male	72 (57.6%)
Female	53 (42.4%)
Location in pancreas, n (%)	
Head/neck	64 (51.2%)
Body/tail	61 (49.8%)
Resectability of pancreatic cancer, n (%)	
Resectable	55 (44.0%)
Borderline resectable	54 (43.2%)
Locally advanced	16 (12.8%)
Histopathologic features of mass, n (%)	
Ductal adenocarcinoma	114 (91.2%)
Adenosquamous carcinoma	9 (7.2%)
Mucinous noncystic carcinoma	1 (0.8%)
Undifferentiated carcinoma	1 (0.8%)
Tumor resection margin status, n (%)	
R0	93 (74.4%)
R1	32 (25.6%)
Y of surgery	2019–2022
N stage, n (%)	
NX	1 (0.8%)
N0	63 (50.4%)
N1	49 (39.2%)
N2	12 (9.6%)
Tumor regression grade, n (%)^a^	
0	3 (2.4%)
1	12 (9.6%)
2	73 (58.4%)
3	37 (29.6%)

*Abbreviations*: *SD* Standard deviation

^a^Grading system of the College of American Pathologists

### CT and MRI examinations

Both the CT and MRI examinations were conducted using pancreatic protocols for all the enrolled patients. For CT examinations, multidetector CT (Toshiba Aquillion One 320, SIEMENS Sensation Cardiac 64, Philips Brilliance iCT 128, United imaging uCT 960 +) with a precontrast scan and 3-phase (arterial phase, parenchymal phase, and delayed phase; contrast agent, 90–95 ml with a rate of 2.5–5.5 ml/s) contrast-enhanced scan was utilized, and the axial section thickness was 1.0/1.5 mm. For MRI examinations, MRI systems (Signa HDxt and MR750, GE Healthcare, Milwaukee, USA; Skyra and Avanto, Siemens, Erlangen, Germany) were used. MRI sequences included coronal and transverse T2-weighted imaging (T2WI), diffusion weighted imaging (DWI) (high b-value 500–1000 s/mm^2^), precontrast T1-weighted imaging (T1WI), and three-phase contrast-enhanced fat-saturated T1-weighted images (contrast agent, 0.1–0.15 mmol/kg with a rate of 2.0–3.0 ml/s).

### Measurement of PC size

In this study, tumor sizes were measured according to the RECIST 1.1 criteria (15). The maximum dimension tumor size described in the radiology reports (CT and MRI) was regarded as the tumor size of the PC. To investigate whether there was difference between the tumor size on radiology reports and the re-measurements, a radiologist who was unaware of the results of pathology reports and radiology reports repeated the measurements of PC sizes both on CT and MRI before and after NAT with 103 patients and 91 patients, respectively. With an interval of six weeks, the radiologist repeated the measurements of PC sizes to evaluate the intraobserver agreement. Evaluation of the target lesions of PC based on pre-NAT CT/MRI and preoperative CT/MRI was performed by the RECIST 1.1 guidelines, and the PC patients were divided into four groups including CR, PR, SD and PD after NAT.

### Pathologic response to NAT

The structured pathological reports for NAT of PC were used in our hospital. Each report recorded the following details: general description, details of materials taken, morphological description, margins and neighbours, lymph node metastasis, diagnosis, tumor regression grade (TRG), etc. The TRG of PC after NAT was performed by the grading system of the College of American Pathologists (CAP), which divides TRG into 4 levels (Grading 0 to 3) according to the ratio of residual tumor cells and the stroma [18]. Grade 0 indicates complete response after NAT of PC and no surviving tumor cells, and grade 3 indicates the NAT was ineffective and many tumor cells remained. The definitions of R0 and R1 were determined according to whether there was absence of tumor cell infiltration within 1 mm of the resection margin. Two pathologists analysed pathological images to issue pathological reports.

### Statistical analysis

Statistical analyses were performed by MedCalc version 13.0.0.0 (MedCalc Software, Ostend, Belgium). The reproducibility between the tumor size on radiology reports and re-measurements and intraobserver agreement of the measurements of PC size were evaluated by using Bland–Altman analyses [19] and intraclass correlation coefficients (ICCs: 0–0.20 = poor correlation, etc.) [20]. Before and after NAT, the differences in the PC tumor sizes between MRI and CT were analysed using the Wilcoxon test (paired samples) with MRI size corrections based on a 5% noninferiority margin. The variability in tumor size measurements of PC on MRI and CT were analyzed by Bland–Altman analyses and ICCs. The differences and variability in tumor sizes between MRI and CT in relationship to different factors, including NAT methods (chemotherapy, chemoradiotherapy), tumor locations (head/neck, body/tail), TRG levels (0–2, 3), N stages (N0, N1/N2) and tumor resection margin status (R0, R1), were further analysed. A receiver operating characteristic (ROC) curve was used to investigate differences in tumor size as measured by CT and MRI to differentiate the tumor resection margin status (R0, R1) and TRG levels (0–2, 3). The discrepancy in NAT evaluations based on the CT and MRI measurements between the groups (PD/SD, CR/PR) was also analysed with the McNemar test (paired samples). The statistical significance levels were set at a *P* value < 0.05.

## Results

### Patients

The characteristics of the 125 patients are detailed in Table 1. There were 72 men and 53 women with a mean age of 60.6 ± 8.5 (33–76), and 51.2% (64 of 125 patients) of the tumors were located in the pancreatic head/neck. Except for one case without peripancreatic lymphadenectomy, metastases to regional lymph nodes were found in 50% (62 of 124 patients) of patients on histological examinations. Among the cases enrolled in this study, there were 55, 54 and 16 patients with resectable, borderline resectable and locally advanced PC, respectively.

### Tumor size measurements between CT and MRI

There were excellent agreements in the PC sizes on radiology reports and re-measurements, with mean size differences and LOAs of -1.5 [-10.4 to 7.4] mm and -0.9 [-12.5 to 10.6] mm for both CT and MRI before NAT (ICCs, 0.95 and 0.92) and -2.1 [-13.9 to 9.7] mm and -2.2 [-17.2 to 12.9] mm after NAT (ICCs, 0.92 and 0.87), respectively. For the twice measurements of PC size by a radiology, there were excellent agreements with mean size differences and LOAs of -0.3 [-6.8 to 6.1] mm and -0.2 [-7.2 to 6.7] mm for both CT and MRI before NAT (ICCs, 0.98 and 0.97) and -0.5 [-7.0 to 6.0] mm and -0.2 [-7.2 to 6.8] mm after NAT (ICCs, 0.98 and 0.97), respectively.

**Fig. 2:**
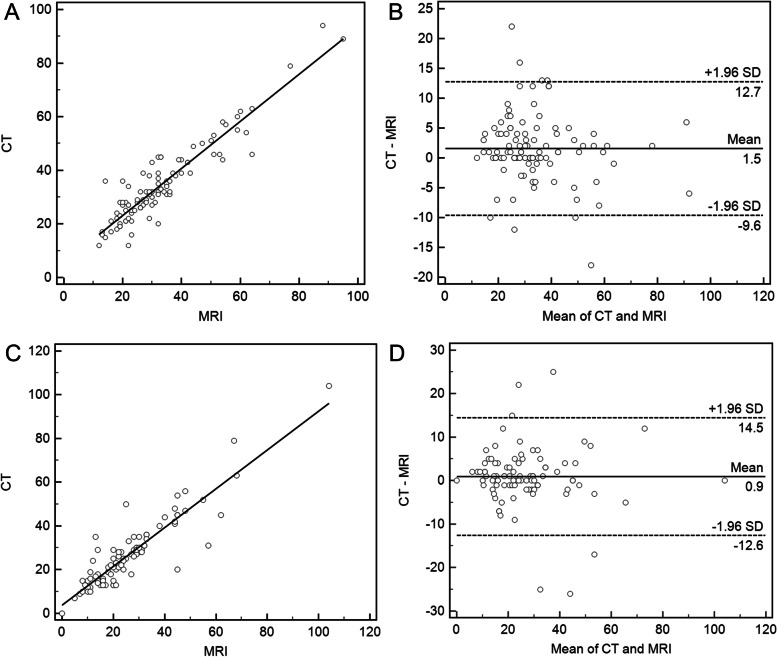
Scatter plots and Bland–Altman plots for tumor size measured by CT and MRI. **A** Scatter plot before neoadjuvant therapy (NAT); **B** Bland‒Altman plot before NAT; **C** Scatter plot after NAT; **D** Bland–Altman plot after NAT

There was no significant difference in the median tumor sizes on CT and MRI before and after NAT with 103 (*P* = 0.44) and 91 (*P* = 0.39) subjects, respectively. There were excellent agreements in the tumor sizes on MRI and CT, with mean size differences and LOAs of 1.5 [-9.6 to 12.7] mm and 0.9 [-12.6 to 14.5] mm before NAT (ICC, 0.93) and after NAT (ICC, 0.91), respectively (Fig. 2). It is worth noting that the median tumor sizes based on preoperative MRI and CT were 22.0 mm and 24.0 mm, respectively, and both the mean CT and MRI sizes of PC were significantly smaller than the pathological size (median, 30.0 mm) (both *P* < 0.001) (Table 2).

**Table 2:** Tumor sizes of preneoadjuvant therapy (NAT) and preoperative MRI, CT and pathology

Parameters	Pre-NAT (*n* = 103)		Preoperative (*n* = 91)		Pathology (*n* = 91)
	MRI	CT	MRI	CT	
Median, mm	30	32	22	24	30
25th percentile, mm	22	26	15	15.5	20
75th percentile, mm	36.5	41	31	32	42
Min, mm	12	12	0	0	5
Max, mm	95	94	104	104	100

For the tumor size measurements of PC on both the pre-NAT and preoperative CT and MRI images given different factors, including NAT methods (chemotherapy, chemoradiotherapy), tumor locations (head/neck, body/tail), TRG levels (0–2, 3), N stages (N0, N1/N2) and tumor resection margin status (R0, R1), there were good or excellent correlations under all the factors, with ICCs ranging from 0.76 to 0.95, the mean size differences ranging from -1.4 mm to 2.5 mm and a range of LOAs for tumor size measurements <  ± 16 mm (Tables 3 and 4); that is, there was good correlation and consistency between CT and MRI tumor sizes in the subgroup analysis mentioned above.

**Table 3:** Comparisons of tumor sizes between MRI and CT before neoadjuvant therapy (NAT) of pancreatic cancer (*n* = 103)

Parameters	NAT methods		Tumor locations		TRG levels		N stages		Resection margin status	
	Chemotherapy (*n* = 69)	Chemoradiotherapy (*n* = 34)	head/neck (*n* = 53)	body/tail (*n* = 50)	0–2 (*n* = 73)	3 (*n* = 30)	N0 (*n* = 51)	N1/N2 (*n* = 51)	R0 (*n* = 76)	R1 (*n* = 27)
Mean size difference, mm	1.6	1.4	1.5	1.6	1.8	0.9	2.5	0.6	2.0	0.2
Limits of agreement, mm	-10.4 to 13.6	-8.1 to 11.0	-9.7 to 12.6	-9.7 to 12.9	-7.7 to 11.3	-13.7 to 15.5	-7.5 to 12.5	-11.6 to 12.7	-9.1 to 13.1	-11.0 to 11.4
ICC	0.93	0.91	0.80	0.95	0.95	0.85	0.94	0.92	0.94	0.89
95% CI of ICC	0.89 to 0.96	0.82 to 0.95	0.68 to 0.88	0.91 to 0.97	0.92 to 0.97	0.71 to 0.93	0.90 to 0.97	0.86 to 0.95	0.90 to 0.96	0.78 to 0.95

*TRG* Tumor regression grade, *ICC* Intraclass correlation coefficient, *CI* Confidence interval

**Table 4:** Comparisons of tumor sizes between MRI and CT after neoadjuvant therapy (NAT) of pancreatic cancer (*n* = 91)

Parameters	NAT methods		Tumor locations		TRG levels		N stages		Resection margin status	
	Chemotherapy (*n* = 61)	Chemoradiotherapy (*n* = 30)	head/neck (*n* = 41)	body/tail (*n* = 50)	0–2 (*n* = 61)	3 (*n* = 30)	N0 (*n* = 42)	N1/N2 (*n* = 48)	R0 (*n* = 69)	R1 (*n* = 22)
Mean size difference, mm	0.9	1.0	0.4	1.4	0.6	1.5	1.4	0.5	1.6	-1.4
Limits of agreement, mm	-14.2 to 15.9	-9.1 to 11.1	-10.9 to 11.6	-13.9 to 16.6	-11.8 to 13.0	-14.3 to 17.3	-12.0 to 14.7	-13.4 to 14.5	-11.3 to 14.6	-16.0 to 13.3
ICC	0.91	0.91	0.81	0.91	0.89	0.90	0.94	0.82	0.93	0.76
95% CI of ICC	0.85 to 0.94	0.81 to 0.95	0.67 to 0.89	0.85 to 0.95	0.82 to 0.93	0.81 to 0.95	0.89 to 0.97	0.71 to 0.90	0.89 to 0.95	0.50 to 0.89

*TRG* Tumor regression grade, *ICC* Intraclass correlation coefficient, *CI* Confidence interval

### Tumor resection margin status and TRG levels in size estimates

**Fig. 3:**
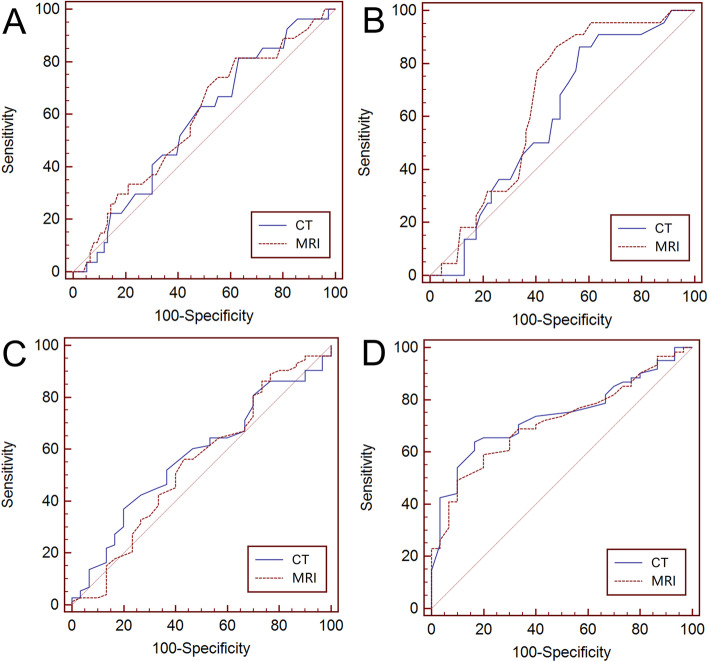
Comparisons of the receiver operating characteristic curves for distinguishing the tumor resection margin status (R0, R1) and tumor regression grade (TRG) levels (0–2, 3) with tumor size measured by CT and MRI. **A** Distinguishing R0 and R1 preneoadjuvant therapy (NAT), *n* = 103; **B** Distinguishing R0 and R1 after NAT, *n* = 91; **C** Distinguishing TRG levels pre-NAT, *n* = 103; D: Distinguishing TRG levels after NAT, *n* = 91

The area under the curve (AUC) was not significantly different for distinguishing between the R0 and R1 groups using tumor sizes with pre-NAT (AUC, 0.57 and 0.58; *P* = 0.64) or preoperative CT and MRI measurements (AUC, 0.60 and 0.66; *P* = 0.08). Additionally, for the comparisons of AUC of the TRG levels, no significant differences were observed between the two groups of TRG with pre-NAT (AUC, 0.57 and 0.54; *P* = 0.42) or preoperative CT and MRI measurements (AUC, 0.74 and 0.72; *P* = 0.48) (Fig. 3).

### Efficacy evaluation of NAT with CT and MRI measurements

Based on tumor sizes from the CT and MRI radiology reports, 77% (60/78) of patients had the same efficacy evaluation group with the guideline of RECIST 1.1. McNemar test (paired samples) results showed no statistically significant difference for the efficacy evaluation of NAT with CT and MRI measurements (*P* = 1.0) (Table 5). With the first measurements of PC sizes of the radiologist, there was about 85% (66/78) of the patients had the same response category group. No significant difference was also found in the efficacy evaluation of NAT between CT and MRI measurements (*P* = 0.15).

**Table 5:** NAT evaluations with the CT and MRI measurements between the groups (PD/SD, CR/PR) based on the RECIST 1.1 guidelines

	*Evaluation of NAT with MRI*		
*Evaluation of NAT with CT*	PD/SD	CR/PR	Total
PD/SD	26	9	35(44.9%)
CR/PR	9	34	43(55.1%)
Total	35(44.9%)	43(55.1%)	78

*Abbreviations*: *NAT* Neoadjuvant therapy, *CR* Complete response, *PR* Partial response, *SD* Stable disease, *PD* Progressive disease

## Discussion

NAT has been a considerable and important treatment strategy for PC patients. It reduces the tumor stage, allows patients with borderline resectable and locally advanced PC to obtain surgical opportunities and increases the rate of negative margins (R0 resection) with NAT [11–14]. After NAT, PC cell damage and NAT-induced tumor interstitial fibrosis are similar to the fibroproliferative matrix of PC itself and pancreatitis fibrosis. Traditional imaging, such as CT and MRI, cannot accurately evaluate the efficacy of NAT because it cannot distinguish between fibrous tissue and cancer tissue after NAT [21, 22]. In addition, it is still difficult to assess whether the tumor shows regression. Nevertheless, both CT and MRI are still the most important tools for the diagnosis and evaluation of PC with treatment [9]. Many international guidelines recommend using CT first in the diagnosis of suspected PC [5, 23–26]. MRI generally shows the same ability of PC detection as CT and may have a potential to reveal structural nature of pancreas more precisely than CT in the patients whose lesions are hard to be recognized in traditional CT.. The RECIST 1.1 method is widely adopted to evaluate the efficacy of solid tumor therapy by measuring the change in tumor size based on CT or MRI before and after treatment. Therefore, it is important to assess PC size with NAT and select the most effective treatment in clinical practice. The importance of tumor size measurement after NAT for PC has been emphasized in the AJR Expert Narrative Panel Review [9]. In this study, our results showed that MRI and CT have similar performance in assessing PC tumor size before and after NAT.

To further confirm our findings, we performed tumor size analyses between CT and MRI measurements in PC patients with NAT by five stratifications, including NAT methods (chemotherapy, chemoradiotherapy), tumor locations (head/neck, body/tail), TRG levels (0–2, 3), N stages (N0, N1/N2) and tumor resection margin status (R0, R1). The results were similar to the conclusions from assessment of the overall cases before and after NAT. In the current study, the average interval between CT and MRI examinations performed preoperatively after NAT was 1.6 days, and the interval between preoperative CT or MRI examinations and the day of operations was 5 days. It is worth noting that the preoperative CT and MRI tumor sizes after NAT were significantly smaller than the pathological size of PC (both *P* < 0.001), with a median tumor size underestimation of approximately 8 mm and 6 mm for MRI and CT, respectively. These findings are similar to our previous studies [27, 28] and those of Arnold et al. [29] and Hall et al. [30], in which resectable PC without NAT had CT or MRI sizes smaller than the pathological sizes. Possible reasons for the tumor CT and MRI sizes of resectable PC being inconsistent with the pathological conclusions have been detailed in our previous reports [27, 28]. In this study, PC was highly dispersed and presented a leap-forward growth pattern. Tumor cells are often distributed in a wide range of stroma, especially around the tumor. In pathological examination, it is usually impossible to distinguish between NAT-induced fibrosis and dense fibrous stroma, so it is difficult to define the tumor contour before and after NAT, and imaging evaluation often underestimates the number of tumor cells scattered around the tumor.

The tumor resection margin status and TRG levels of PC are important clinical indicators for NAT. In the current study, there were no significant differences in distinguishing between the R0 and R1 groups or TRG levels using tumor sizes from pre-NAT or preoperative CT and MRI measurements. Additionally, there was no significant difference in the efficacy evaluation of NAT between CT and MRI measurements, and the measurements of PC sizes by the same radiology before and after NAT will improve the accuracy of response category between CT and MRI. These results further confirm that MRI and CT have similar performance in assessing PC tumor size after NAT.

Tumor size and tumor stage after NAT are key indicators for evaluating efficacy. Our study has compared the difference between the imaging and pathological sizes of tumors after NAT for PC. In pathological analysis after NAT for PC, the specification of pathological sampling has not been unified. In this study, the pathological diagnosis of 3 cases was complete regression after NAT, but nodular-like mass could still be observed in gross, and there were also corresponding imaging findings on CT and MRI. Traditional pathological small sections have errors in measuring the size of PC after NAT, and whole-mount pathological analyses will have better application prospects in T staging [31]. Multidisciplinary clinics are very important for NAT for PC. It is expected that there will be standardized methods developed from imaging examination, follow-up during treatment and pathology analysis after NAT to provide more accurate strategies for treatment of PC patients and to improve treatment effects.

Our study had some limitations. First, this study was designed as a retrospective study; the aspects that we could control were limited. Second, the neoadjuvant methods used in this study were not uniform. For example, FOLFIRINOX, mFOLFIRINOX, albumin-bound paclitaxel plus gemcitabine were used in chemotherapy. Due to sample size limitations, we did not further stratify different chemotherapy regimens.

## Conclusion

Both CT and MRI have significant advantages in the assessment of PC. Our findings showed that MRI and CT have similar efficacy in the assessment of tumor size before and after NAT for PC, and MRI may have more advantages in multiple follow-ups because of the absence of ionizing radiation. Therefore, the guidelines and application value of PC size assessment after NAT need to be further clarified and studied to make better treatment decisions in clinical practice.

## Supplementary Information

**Additional file 1.**

## Data Availability

The research dataset for the current study is available from the corresponding author upon reasonable request.

## Abbreviations

CT: Computed tomography
MRI: Magnetic resonance imaging
PC: Pancreatic cancer
NAT: Neoadjuvant therapy
ICC: Intraclass correlation coefficient
TRG: Tumor regression grade
RECIST: Response evaluation criteria in solid tumors
LOA: Limits of agreement
CR: Complete response
PR: Partial response
SD: Stable disease
PD: Progressive disease
ROC: Receiver operating characteristic
AUC: Area under the curve
