# Supplementary material for: Tumor size measurements of pancreatic cancer with neoadjuvant therapy based on RECIST guidelines: is MRI as effective as CT?
Source: Cancer Imaging. 2023 Jan 18;23:8. doi: 10.1186/s40644-023-00528-z (PMC9850516; doi:10.1186/s40644-023-00528-z)
Supplement: Supplementary file 1 — Additional file 1. [file 40644_2023_528_MOESM1_ESM.docx]

**Response to reviewers**

We thank the reviewers and editor for their encouragement. We have made systematic revisions addressing each of the comments. To facilitate the re-review process, the modifications are introduced in the following response letter and are marked in the revised manuscript, along with the annotations in the margin numbered by the reviewers’ comments (for example, R1C1 indicates our change addressing Reviewer1’s Comment 1). We hope that the revised revision will meet the reviewers’ expectations and the publication standards of *Cancer Imaging*.

**Reviewer #1:**

*Comment R1C1: General comments: Despite previous our pointing outs, additional data or explanations are not added in this manuscript, especially as to PC size measurement and intraobserver agreement. As you described in discussion, MRI may have a potential to reveal structural nature of pancreas more precisely than CT, but I cannot help doubting authors statement that MRI (T1-weighted) images generally show same ability of PC detection as CT images. I think authors statement would be accepted in the limited patients whose lesions are hard to be recognized in traditional CT.*

Response R1C1: We greatly appreciate the reviewer’s overall comments and suggestions. To investigate the intraobserver agreement for the measurements of PC sizes, a radiologist performed twice measurements of PC sizes both on CT and MRI before and after NAT with 103 patients and 91 patients with an interval of 6 weeks, respectively. For the twice measurements of PC sizes, there were excellent agreements with mean size differences and LOAs of -0.3 [-6.8 to 6.1] mm and -0.2 [-7.2 to 6.7] mm for both CT and MRI before NAT (ICCs, 0.98 and 0.97) and -0.5 [-7.0 to 6.0] mm and -0.2 [-7.2 to 6.8] mm after NAT (ICCs, 0.98 and 0.97), respectively. According to the reviewer’s comments and suggestions, modifications were made as recommended. MRI generally shows the same ability of PC detection as CT and may have a potential to reveal structural nature of pancreas more precisely than CT in the patients whose lesions are hard to be recognized in traditional CT.

*Comment R1C2: Specific Comments: Minor comment: There are still some incorrect descriptions as to statistically analyzed data.*

Response R1C2: We thank the reviewer for the careful review and have made a careful revision addressing issues in the descriptions as to statistically analyzed data.

**Reviewer #2:**

General comments:

*The authors have addressed all of the issues raised by the reviewers and solved most of them. Formatting and language improved. However, some important issues have still to be addressed*

Response: We greatly appreciate the reviewer’s overall comments.

Specific Comments:

*Comment R2C1："Tumor size measurements between CT and MRI There were excellent agreements in the PC sizes on radiology reports and re-measurements, with mean size differences and LOAs of -1.5 [-10.4-7.4] mm and -0.9 [-12.5-10.6] mm for both CT and MRI before NAT (ICCs, 0.95 and 0.92) and -2.1 [-13.9-9.7] mm and -2.2 [-17.2-12.9] mm ..." -12.5-10.6 mm is confusing for the readers! you have to format differently: -12.5 ─ 10.6 mm*

Response R2C1: We thank the reviewer for the suggestions. and modifications were made to make the contents more concise. E.g., -12.5 to 10.6 mm.

*Comment R2C2：*

*"Tumor Size Measurements of Pancreatic Cancer with Neoadjuvant Therapy Based on RECIST Guidelines: Is MRI as Effective as CT?" Actually the chemotherapy will be effective (or not), but the size measurements can only be accurate or not.*

Response R2C2: We thank the reviewer for the comments. Yes, this is right.

*Comment R2C3： Abstract:*

*"There was no significant difference in the efficacy evaluation of NAT between CT and MRI measurements (P=1.0), which showed that 77% of patients had the same grade."*

*What do the authors means by grade? Obviously the same response category (as shown in the new Table 5)? I dont´think that an agreement between CT and MRI about the chemotherapy response criteria in only 77% of patients can be left without further comment on the side of the authors. It means that in 1 of 4 patients actually CT and MRI methods disagreed.*

Response R2C3: We thank the reviewer for the comments. We have revised the incorrect descriptions as to statistically analyzed data. The NAT evaluations with the CT and MRI measurements between two groups (PD/SD, CR/PR) based on the RECIST 1.1 criteria was analyzed using McNemar test. There was no significant difference in the efficacy evaluation of NAT between CT and MRI measurements (P=1.0) when using the tumor size on radiology reports, and there was about 77% of the patients had the same response category group. To investigate the intraobserver agreement for the measurements of PC sizes, a radiologist performed twice measurements of PC sizes both on CT and MRI before and after NAT with 103 patients and 91 patients with an interval of 6 weeks, respectively. With the first measurements of PC sizes by the radiologist, there was about 85% of the patients had the same response category group. The measurements of PC size from radiology reports which were issued by different radiologists may be closer to the real clinical practice, but the measurements of PC size by the same radiology before and after NAT will improve the accuracy of response category between CT and MRI. We have revised the manuscript accordingly to make the content more concise.

*Comment R2C4："contrast agent, 90-95 ml with a rate of 2.5-5.5 ml/s)"*

*This data leave the reader a little bit puzzled. Why do all the patients get the same amount of contrast material (at which concentration?), but with such a high variation of the flow rate? Please comment.*

*"(contrast agent, 0.2-0.3 ml/kg with a rate of 2.0-3.0 ml/s)."*

*For MRI better give mmol/kg or you have to specify the concentration ..*

Response R2C4: We thank the reviewer for the careful review. The CT contrast agent (90-95 mL, Ultravist 370; Bayer Schering Pharma) was injected at a rate of 2.5-5.5 mL/sec with a power injector via the forearm vein. Before CT or MRI examinations, our nurses evaluate each patient's vascular condition and give feedback to the imaging technicians, who empirically selects the contrast injection rate to avoid breaking the blood vessels and causing contrast extravasation while imaging. So, such a high variation of the flow rate was used. Contrast-enhanced MRI were performed with gadopentetate dimeglumine injection (contrast agent, 0.1-0.15 mmol/kg with a rate of 2.0-3.0 ml/s).

*Comment R2C5：*

*"There was no significant difference in the mean tumor sizes on CT and MRI before and after NAT with 103 (P = 0.44) and 91 (P = 0.39) subjects, respectively."*

*Still the mean tumor size instead of the median is mentioned.*

*"the median tumor sizes based on preoperative MRI and CT were 22.0 mm and 24.0 mm, mrespectively, " (spelling error)*

*Importantly, to make the life of the reviewer easier:*

*Submit one copy with letter together with the annotated manuscript (incl Tables, etc.) plus one clean manuscript (incl Tables, etc.) in the Supplemental material.*

Response R2C5: We thank the reviewer for the careful review and modifications were made in the revised manuscript. A file of response letter together with the annotated manuscript (including Tables, etc.) plus one clean manuscript (including Tables, etc.) was also submitted in the Supplemental material.

**Marked Manuscript CAIG-D-22-00333R1**

**Tumor Size Measurements of Pancreatic Cancer with Neoadjuvant Therapy Based on RECIST Guidelines: Is MRI as Effective as CT?**

Panpan Yang^1,#^, Kuanzheng Mao^1,2,#^, Yisha Gao^3^, Zhen Wang^1^, Jun Wang^1^, Yufei Chen^4^, Chao Ma^1,4,*^, Yun Bian^1^, Chengwei Shao^1^, Jianping Lu^1,*^

^1^Department of Radiology, Changhai Hospital of Shanghai, Naval Medical University, China; ^2^School of Health Science and Engineering, University of Shanghai for Science and Technology, China; ^3^Department of Pathology, Changhai Hospital of Shanghai, Naval Medical University, China; ^4^College of Electronic and Information Engineering, Tongji University, Shanghai, China

**E-mail of authors:**

Panpan Yang, panwang1112@163.com; Kuanzheng Mao, 541071673@qq.com; Yisha Gao, 241177536@qq.com; Zhen Wang, 314252293@qq.com; Jun Wang, wangjun_ying@sina.com; Yufei Chen, april337@163.com; Chao Ma, mengqihi@gmail.com; Yun Bian, bianyun2012@foxmail.com; Chengwei Shao, chengweishaoch@163.com; Jianping Lu, cjr.lujianping@vip.163.com

**Corresponding to:** Department of Radiology, Changhai Hospital of Shanghai, Naval Medical University, No. 168 Changhai Road, Shanghai 200433, China. cjr.lujianping@vip.163.com (Jianping Lu), mengqihi@gmail.com (Chao Ma)

^#^These authors contributed equally to this work.

**Manuscript Type:** Original research

**Abstract**

**Objectives:** To compare tumor size measurements using CT and MRI in pancreatic cancer (PC) patients with neoadjuvant therapy (NAT).

**Methods:** This study included 125 histologically confirmed PC patients who underwent NAT. The tumor sizes from CT and MRI before and after NAT were compared by using Bland-Altman analyses and intraclass correlation coefficients (ICCs). Variations in tumor size estimates between MRI and CT in relationship to different factors, including NAT methods (chemotherapy, chemoradiotherapy), tumor locations (head/neck, body/tail), tumor regression grade (TRG) levels (0-2, 3), N stages (N0, N1/N2) and tumor resection margin status (R0, R1), were further analysed. The McNemar test was used to compare the efficacy of NAT evaluations based on the CT and MRI measurements according to RECIST 1.1 criteria.

R1C2

R2C5

**Results:** There was no significant difference between the median tumor sizes from CT and MRI before and after NAT (P=0.44 and 0.39, respectively). There was excellent agreement in tumor size between MRI and CT, with mean size differences and limits of agreement (LOAs) of 1.5 [-9.6 to 12.7] mm and 0.9 [-12.6 to 14.5] mm before NAT (ICC, 0.93) and after NAT (ICC, 0.91), respectively. For all the investigated factors, there was good or excellent correlation (ICC, 0.76 to 0.95) for tumor sizes between CT and MRI. There was no significant difference in the efficacy evaluation of NAT between CT and MRI measurements (P=1.0).

R2C3

R1C2

R2C1

**Conclusion:** MRI and CT have similar performance in assessing PC tumor size before and after NAT.

**Keywords:** Pancreatic cancer; neoadjuvant therapy, CT; MRI; tumor size

**Introduction**

As a lethal cancer with poor prognosis and high mortality, pancreatic cancer (PC) results in more than 460,000 deaths a year worldwide [1] and may be the second leading cause of death due to cancer by 2030 in the United States [2]. According to the cancer statistics of the United States in 2022, the 5-year survival rate of PC is only 11% [3]. Despite surgical resection is the possible cure method for PC, nowadays the therapeutic principle clearly is not always operation first, considering neoadjuvant therapy (NAT). Most patients with PC have local tumor progression or distant metastasis at diagnosis and miss the opportunity for surgical resection. Only approximately 10%-15% of the patients who are evaluated as having resectable PC can undergo radical surgical resection. More patients are diagnosed with borderline resectable or locally advanced PC [4]. To improve the management of this lethal tumor, on the one hand, earlier diagnosis of resectable PC should be explored, while, on the other hand, new treatment methods should be developed.

NAT is a treatment strategy for resectable, borderline resectable and locally advanced PC that has been emerging considerably [5-9]. The definition of NAT for PC is preoperative chemotherapy with or without radiation therapy [10]. The purpose of NAT for PC is to reduce the tumor stage, improve the rate of R0 resection (a microscopically margin-negative resection), and reduce postoperative recurrence and metastasis [11-14]. Ideal methods for reassessment after NAT for PC are lacking, and currently, the response evaluation criteria in solid tumors (RECIST) are still the main choice for use [9,15]. Investigators could adopt RECIST criteria to assess the treatment outcomes including complete response (CR), partial response (PR), stable disease (SD) and progressive disease (PD) after NAT for PC patients with tumor size measurements. CT and MRI are the commonly used imaging modalities for the diagnosis and evaluation of PC and show similar effects in the staging and diagnosis of pancreatic cancer, both pre-NAT and preoperation without NAT [16]. Different from other solid tumors, the changes in tissue components of PC after NAT, including tumor necrosis, oedema, inflammation, and fibrosis, result in significant effects on CT-image evaluations of tumor treatment and resectability [17]. The accurate staging of tumors with CT or MRI is used not only for the selection of treatment methods but also for accurate tumor measurement to improve outcomes in PC patients after NAT in clinical practice. In this study, we investigated the similarities and differences in tumor measurements between CT and MRI before and after NAT for PC based on RECIST 1.1 criteria.

**Methods**

**Patients**

Our institutional review board approved this retrospective study. We undertook a review of all patients who had undergone pancreatic contrast-enhanced MRI or contrast-enhanced CT and pancreatic tumor resection between April 2019 and December 2021 and identified 1752 pancreatic tumor patients. Among these patients, 125 subjects (72 males, 53 females; mean age: 60.6 ± 8.5 years; range: 33-76 years) had received NAT and had detailed pathological reports. Before NAT, 104 patients underwent both contrast-enhanced CT and contrast-enhanced MRI examinations within an interval of less than 14 days. After NAT, a total of 109 patients underwent both preoperative contrast-enhanced CT and contrast-enhanced MRI examinations, and 17 patients were excluded due to an MRI or CT examination time more than 14 days before the day of the operation. Additionally, a patient with tumor sizes less than 10 mm per pre-NAT CT and MRI measurements was excluded. Finally, this study included 103 and 91 patients who had received both CT and MRI examinations in the pre-NAT group and the finished-NAT group, respectively. Among the 125 enrolled cases, a total of 78 patients who underwent both CT and MRI within an interval of less than 14 days before NAT and subsequently underwent preoperative CT and MRI less than 14 days before the day of the operation were chosen for further analyses (Figure 1, Table 1).

**CT and MRI examinations**

Both the CT and MRI examinations were conducted using pancreatic protocols for all the enrolled patients. For CT examinations, multidetector CT (Toshiba Aquillion One 320, SIEMENS Sensation Cardiac 64, Philips Brilliance iCT 128, United imaging uCT 960+) with a precontrast scan and 3-phase (arterial phase, parenchymal phase, and delayed phase; contrast agent, 90-95 ml with a rate of 2.5-5.5 ml/s) contrast-enhanced scan was utilized, and the axial section thickness was 1.0/1.5 mm. For MRI examinations, 3.0-T MRI systems (Signa HDxt and MR750, GE Healthcare, Milwaukee, USA; Skyra, Siemens, Erlangen, Germany) were used. MRI sequences included coronal and transverse T2-weighted imaging (T2WI), diffusion weighted imaging (DWI) (high b-value 500-1000 s/mm^2^), precontrast T1-weighted imaging (T1WI), and three-phase contrast-enhanced fat-saturated T1-weighted images (contrast agent, 0.1-0.15 mmol/kg with a rate of 2.0-3.0 ml/s).

R2C4

**Measurement of PC size**

In this study, tumor sizes were measured according to the RECIST 1.1 criteria (15). The maximum dimension tumor size described in the radiology reports (CT and MRI) was regarded as the tumor size of the PC. To investigate whether there was difference between the tumor size on radiology reports and the re-measurements, a radiologist who was unaware of the results of pathology reports and radiology reports repeated the measurements of PC sizes both on CT and MRI before and after NAT with 103 patients and 91 patients, respectively. With an interval of six weeks, the radiologist repeated the measurements of PC sizes to evaluate the intraobserver agreement. Evaluation of the target lesions of PC based on pre-NAT CT/MRI and preoperative CT/MRI was performed by the RECIST 1.1 guidelines, and the PC patients were divided into four groups including CR, PR, SD and PD after NAT.

R1C1

**Pathologic response to NAT**

The structured pathological reports for NAT of PC were used in our hospital. Each report recorded the following details: general description, details of materials taken, morphological description, margins and neighbours, lymph node metastasis, diagnosis, tumor regression grade (TRG), etc. The TRG of PC after NAT was performed by the grading system of the College of American Pathologists (CAP), which divides TRG into 4 levels (Grading 0 to 3) according to the ratio of residual tumor cells and the stroma [18]. Grade 0 indicates complete response after NAT of PC and no surviving tumor cells, and grade 3 indicates the NAT was ineffective and many tumor cells remained. The definitions of R0 and R1 were determined according to whether there was absence of tumor cell infiltration within 1 mm of the resection margin. Two pathologists analysed pathological images to issue pathological reports.

**Statistical analysis**

Statistical analyses were performed by MedCalc version 13.0.0.0 (MedCalc Software, Ostend, Belgium). The reproducibility between the tumor size on radiology reports and re-measurements and intraobserver agreement of the measurements of PC size were evaluated by using Bland-Altman analyses (19) and intraclass correlation coefficients (ICCs: 0-0.20 = poor correlation, etc.) (20). Before and after NAT, the differences in the PC tumor sizes between MRI and CT were analysed using the Wilcoxon test (paired samples) with MRI size corrections based on a 5% noninferiority margin. The variability in tumor size measurements of PC on MRI and CT were analyzed by Bland-Altman analyses and ICCs. The differences and variability in tumor sizes between MRI and CT in relationship to different factors, including NAT methods (chemotherapy, chemoradiotherapy), tumor locations (head/neck, body/tail), TRG levels (0-2, 3), N stages (N0, N1/N2) and tumor resection margin status (R0, R1), were further analysed. A receiver operating characteristic (ROC) curve was used to investigate differences in tumor size as measured by CT and MRI to differentiate the tumor resection margin status (R0, R1) and TRG levels (0-2, 3). The discrepancy in NAT evaluations based on the CT and MRI measurements between the groups (PD/SD, CR/PR) was also analysed with the McNemar test (paired samples). The statistical significance levels were set at a P value < 0.05.

R1C1

**Results**

**Patients**

The characteristics of the 125 patients are detailed in Table 1. There were 72 men and 53 women with a mean age of 60.6 ± 8.5 (33-76), and 51.2% (64 of 125 patients) of the tumors were located in the pancreatic head/neck. Except for one case without peripancreatic lymphadenectomy, metastases to regional lymph nodes were found in 50% (62 of 124 patients) of patients on histological examinations. Among the cases enrolled in this study, there were 55, 54 and 16 patients with resectable, borderline resectable and locally advanced PC, respectively.

**Tumor size measurements between CT and MRI**

There were excellent agreements in the PC sizes on radiology reports and re-measurements, with mean size differences and LOAs of -1.5 [-10.4 to 7.4] mm and -0.9 [-12.5 to 10.6] mm for both CT and MRI before NAT (ICCs, 0.95 and 0.92) and -2.1 [-13.9 to 9.7] mm and -2.2 [-17.2 to 12.9] mm after NAT (ICCs, 0.92 and 0.87), respectively. For the twice measurements of PC size by a radiology, there were excellent agreements with mean size differences and LOAs of -0.3 [-6.8 to 6.1] mm and -0.2 [-7.2 to 6.7] mm for both CT and MRI before NAT (ICCs, 0.98 and 0.97) and -0.5 [-7.0 to 6.0] mm and -0.2 [-7.2 to 6.8] mm after NAT (ICCs, 0.98 and 0.97), respectively.

R1C1

There was no significant difference in the median tumor sizes on CT and MRI before and after NAT with 103 (P = 0.44) and 91 (P = 0.39) subjects, respectively. There were excellent agreements in the tumor sizes on MRI and CT, with mean size differences and LOAs of 1.5 [-9.6 to 12.7] mm and 0.9 [-12.6 to 14.5] mm before NAT (ICC, 0.93) and after NAT (ICC, 0.91), respectively (Figure 2). It is worth noting that the median tumor sizes based on preoperative MRI and CT were 22.0 mm and 24.0 mm, respectively, and both the mean CT and MRI sizes of PC were significantly smaller than the pathological size (median, 30.0 mm) (both P < 0.001) (Table 2).

R2C5

R1C2

R2C1

For the tumor size measurements of PC on both the pre-NAT and preoperative CT and MRI images given different factors, including NAT methods (chemotherapy, chemoradiotherapy), tumor locations (head/neck, body/tail), TRG levels (0-2, 3), N stages (N0, N1/N2) and tumor resection margin status (R0, R1), there were good or excellent correlations under all the factors, with ICCs ranging from 0.76 to 0.95, the mean size differences ranging from -1.4 mm to 2.5 mm and a range of LOAs for tumor size measurements < ± 16 mm (Tables 3 and 4); that is, there was good correlation and consistency between CT and MRI tumor sizes in the subgroup analysis mentioned above.

**Tumor resection margin status and TRG levels in size estimates**

The area under the curve (AUC) was not significantly different for distinguishing between the R0 and R1 groups using tumor sizes with pre-NAT (AUC, 0.57 and 0.58; P = 0.64) or preoperative CT and MRI measurements (AUC, 0.60 and 0.66; P = 0.08). Additionally, for the comparisons of AUC of the TRG levels, no significant differences were observed between the two groups of TRG with pre-NAT (AUC, 0.57 and 0.54; P = 0.42) or preoperative CT and MRI measurements (AUC, 0.74 and 0.72; P = 0.48) (Figure 3).

**Efficacy evaluation of NAT with CT and MRI measurements**

Based on tumor sizes from the CT and MRI radiology reports, 77% (60/78) of patients had the same efficacy evaluation group with the guideline of RECIST 1.1. McNemar test (paired samples) results showed no statistically significant difference for the efficacy evaluation of NAT with CT and MRI measurements (P = 1.0) (Table 5). With the first measurements of PC sizes of the radiologist, there was about 85% (66/78) of the patients had the same response category group. No significant difference was also found in the efficacy evaluation of NAT between CT and MRI measurements (P = 0.15).

R2C3

**Discussion**

NAT has been a considerable and important treatment strategy for PC patients. It reduces the tumor stage, allows patients with borderline resectable and locally advanced PC to obtain surgical opportunities and increases the rate of negative margins (R0 resection) with NAT [11-14]. After NAT, PC cell damage and NAT-induced tumor interstitial fibrosis are similar to the fibroproliferative matrix of PC itself and pancreatitis fibrosis. Traditional imaging, such as CT and MRI, cannot accurately evaluate the efficacy of NAT because it cannot distinguish between fibrous tissue and cancer tissue after NAT [21,22]. In addition, it is still difficult to assess whether the tumor shows regression. Nevertheless, both CT and MRI are still the most important tools for the diagnosis and evaluation of PC with treatment [9]. Many international guidelines recommend using CT first in the diagnosis of suspected PC [5,23-26]. MRI generally shows the same ability of PC detection as CT and may have a potential to reveal structural nature of pancreas more precisely than CT in the patients whose lesions are hard to be recognized in traditional CT.. The RECIST 1.1 method is widely adopted to evaluate the efficacy of solid tumor therapy by measuring the change in tumor size based on CT or MRI before and after treatment. Therefore, it is important to assess PC size with NAT and select the most effective treatment in clinical practice. The importance of tumor size measurement after NAT for PC has been emphasized in the AJR Expert Narrative Panel Review [9]. In this study, our results showed that MRI and CT have similar performance in assessing PC tumor size before and after NAT.

R1C1

To further confirm our findings, we performed tumor size analyses between CT and MRI measurements in PC patients with NAT by five stratifications, including NAT methods (chemotherapy, chemoradiotherapy), tumor locations (head/neck, body/tail), TRG levels (0-2, 3), N stages (N0, N1/N2) and tumor resection margin status (R0, R1). The results were similar to the conclusions from assessment of the overall cases before and after NAT. In the current study, the average interval between CT and MRI examinations performed preoperatively after NAT was 1.6 days, and the interval between preoperative CT or MRI examinations and the day of operations was 5 days. It is worth noting that the preoperative CT and MRI tumor sizes after NAT were significantly smaller than the pathological size of PC (both P < 0.001), with a median tumor size underestimation of approximately 8 mm and 6 mm for MRI and CT, respectively. These findings are similar to our previous studies [27,28] and those of Arnold et al [29] and Hall et al [30], in which resectable PC without NAT had CT or MRI sizes smaller than the pathological sizes. Possible reasons for the tumor CT and MRI sizes of resectable PC being inconsistent with the pathological conclusions have been detailed in our previous reports [27,28]. In this study, PC was highly dispersed and presented a leap-forward growth pattern. Tumor cells are often distributed in a wide range of stroma, especially around the tumor. In pathological examination, it is usually impossible to distinguish between NAT-induced fibrosis and dense fibrous stroma, so it is difficult to define the tumor contour before and after NAT, and imaging evaluation often underestimates the number of tumor cells scattered around the tumor.

The tumor resection margin status and TRG levels of PC are important clinical indicators for NAT. In the current study, there were no significant differences in distinguishing between the R0 and R1 groups or TRG levels using tumor sizes from pre-NAT or preoperative CT and MRI measurements. Additionally, there was no significant difference in the efficacy evaluation of NAT between CT and MRI measurements, and the measurements of PC sizes by the same radiology before and after NAT will improve the accuracy of response category between CT and MRI. These results further confirm that MRI and CT have similar performance in assessing PC tumor size after NAT.

R2C3

Tumor size and tumor stage after NAT are key indicators for evaluating efficacy. Our study has compared the difference between the imaging and pathological sizes of tumors after NAT for PC. In pathological analysis after NAT for PC, the specification of pathological sampling has not been unified. In this study, the pathological diagnosis of 3 cases was complete regression after NAT, but nodular-like mass could still be observed in gross, and there were also corresponding imaging findings on CT and MRI. Traditional pathological small sections have errors in measuring the size of PC after NAT, and whole-mount pathological analyses will have better application prospects in T staging [31]. Multidisciplinary clinics are very important for NAT for PC. It is expected that there will be standardized methods developed from imaging examination, follow-up during treatment and pathology analysis after NAT to provide more accurate strategies for treatment of PC patients and to improve treatment effects.

Our study had some limitations. First, this study was designed as a retrospective study; the aspects that we could control were limited. Second, the neoadjuvant methods used in this study were not uniform. For example, FOLFIRINOX, mFOLFIRINOX, albumin-bound paclitaxel plus gemcitabine were used in chemotherapy. Due to sample size limitations, we did not further stratify different chemotherapy regimens.

**Conclusion**

Both CT and MRI have significant advantages in the assessment of PC. Our findings showed that MRI and CT have similar efficacy in the assessment of tumor size before and after NAT for PC, and MRI may have more advantages in multiple follow-ups because of the absence of ionizing radiation. Therefore, the guidelines and application value of PC size assessment after NAT need to be further clarified and studied to make better treatment decisions in clinical practice.

**Abbreviations**

CT: computed tomography; MRI: magnetic resonance imaging; PC: pancreatic cancer; NAT: neoadjuvant therapy; ICC: intraclass correlation coefficient; TRG: tumor regression grade; RECIST: response evaluation criteria in solid tumors; LOA: limits of agreement; CR: complete response, PR: partial response, SD: stable disease; PD: progressive disease; ROC: receiver operating characteristic; AUC: area under the curve

**Acknowledgements**

We thank Mr. Wang Lizhi for his help in data statistics and analysis.

**Authors’ contributions**

Yang P, Mao K, Gao Y, Wang Z, Wang J, Ma C, and Bian Y performed the majority of experiments, made substantial contributions to the data analysis and interpretation, and wrote the manuscript draft; Ma C, Chen Y, Shao C and Lu J participated in the design of the study and made substantial contribution to data analysis; Ma C and Lu J made substantial contributions to the study conception and design, critically revised the manuscript draft for important intellectual content, and gave final approval of the version to be published; all the authors read and approved the final manuscript.

**Funding**

This work was supported by the National Natural Science Foundation of China [82171915, 82171930, 62173252]; 234 Platform Discipline Consolidation Foundation Project of Changhai Hospital [2020YPT001]; Shanghai Science and Technology Innovation Action Plan Medical Innovation Research Project [20Y11912500].

**Data availability**

The research dataset for the current study is available from the corresponding author upon reasonable request.

**Declarations
Ethics approval and consent to participate**

This study was approved by our Institutional Review Board (Shanghai Changhai Hosptial Ethics committee). Signed written informed consent was waived from all participants.

**Consent for publication**

Not applicable.

**Competing interests**

The authors declare that they have no competing interests.

**References**

1. Sung H, Ferlay J, Siegel RL, Laversanne M, Soerjomataram I, Jemal A, et al. Global Cancer Statistics 2020: GLOBOCAN estimates of incidence and mortality worldwide for 36 cancers in 185 Countries. CA Cancer J Clin 2021;71(3):209-249.
2. Saad AM, Turk T, Al-Husseini MJ, Abdel-Rahman O. Trends in pancreatic adenocarcinoma incidence and mortality in the United States in the last four decades; a SEER-based study. BMC Cancer 2018;18(1):688.
3. Siegel RL, Miller KD, Fuchs HE, Jemal A. Cancer statistics, 2022. CA Cancer J Clin 2022;72(1):7-33.
4. Park W, Chawla A, O'Reilly EM. Pancreatic Cancer: A Review. JAMA 2021;326(9):851-862.
5. Tempero MA, Malafa MP, Al-Hawary M, Behrman SW, Benson AB, Cardin DB, et al. Pancreatic adenocarcinoma, Version 2.2021, NCCN clinical practice guidelines in oncology. J Natl Compr Canc Netw 2021;19(4):439-457.
6. Kulkarni NM, Soloff EV, Tolat PP, Sangster GP, Fleming JB, Brook OR, et al. White paper on pancreatic ductal adenocarcinoma from society of abdominal radiology's disease-focused panel for pancreatic ductal adenocarcinoma: Part I, AJCC staging system, NCCN guidelines, and borderline resectable disease. Abdom Radiol (NY) 2020;45(3):716-728.
7. Gugenheim J, Crovetto A, Petrucciani N. Neoadjuvant therapy for pancreatic cancer. Updates Surg 2022;74(1):35-42.
8. Versteijne E, van Dam JL, Suker M, Janssen QP, Groothuis K, Akkermans-Vogelaar JM, et al. Neoadjuvant chemoradiotherapy versus upfront surgery for resectable and borderline resectable pancreatic cancer: Long-term results of the dutch randomized PREOPANC trial. J Clin Oncol 2022;40(11):1220-1230.
9. Soloff EV, Al-Hawary MM, Desser TS, Fishman EK, Minter RM, Zins M. Imaging assessment of pancreatic cancer resectability after neoadjuvant therapy: *AJR* Expert Panel Narrative Review. AJR Am J Roentgenol 2022;218(4):570-581.
10. Heinrich S, Lang H. Neoadjuvant therapy of pancreatic cancer: Definitions and benefits. Int J Mol Sci 2017;18(8):1622.
11. Reni M, Balzano G, Zanon S, Zerbi A, Rimassa L, Castoldi R, et al. Safety and efficacy of preoperative or postoperative chemotherapy for resectable pancreatic adenocarcinoma (PACT-15): a randomised, open-label, phase 2-3 trial. Lancet Gastroenterol Hepatol 2018;3(6):413-423.
12. Townend P, de Reuver PR, Chua TC, Mittal A, Clark SJ, Pavlakis N, et al. Histopathological tumor viability after neoadjuvant chemotherapy influences survival in resected pancreatic cancer: analysis of early outcome data. ANZ J Surg 2018;88(3):E167-E172.
13. Versteijne E, Vogel JA, Besselink MG, Busch ORC, Wilmink JW, Daams JG, et al. Meta-analysis comparing upfront surgery with neoadjuvant treatment in patients with resectable or borderline resectable pancreatic cancer. Br J Surg 2018;105(8):946-958.
14. Versteijne E, Suker M, Groothuis K, Akkermans-Vogelaar JM, Besselink MG, Bonsing BA, et al. Preoperative chemoradiotherapy versus immediate surgery for resectable and borderline resectable pancreatic cancer: results of the dutch randomized phase III PREOPANC trial. J Clin Oncol 2020;38(16):1763-1773.
15. Schwartz LH, Litière S, de Vries E, Ford R, Gwyther S, Mandrekar S, et al. RECIST 1.1-Update and clarification: From the RECIST committee. Eur J Cancer 2016;62:132-7.
16. Treadwell JR, Zafar HM, Mitchell MD, Tipton K, Teitelbaum U, Jue J. Imaging tests for the diagnosis and staging of pancreatic adenocarcinoma: A meta-analysis. Pancreas 2016;45(6):789-95.
17. White RR, Paulson EK, Freed KS, Keogan MT, Hurwitz HI, Lee C, et al. Staging of pancreatic cancer before and after neoadjuvant chemoradiation. J Gastrointest Surg 2001;5(6):626-33.
18. Washington MK, Berlin J, Branton P, Burgart LJ, Carter DK, Fitzgibbons PL, et al. Protocol for the examination of specimens from patients with primary carcinoma of the colon and rectum. Arch Pathol Lab Med 2009;133(10):1539-51.
19. Bland JM, Altman DG. Statistical methods for assessing agreement between two methods of clinical measurement. Lancet 1986; 1(8476):307-10.
20. Cohen J. Weighted kappa: nominal scale agreement with provision for scaled disagreement or partial credit. Psychol Bull 1968;70(4):213-20.
21. Verbeke CS, Knapp J, Gladhaug IP. Tumor growth is more dispersed in pancreatic head cancers than in rectal cancer: implications for resection margin assessment. Histopathology 2011;59(6):1111-21.
22. Wang H, Zhao Q. Pathologic complete response to neoadjuvant therapy in patients with pancreatic ductal adenocarcinoma is associated with better prognosis. Ann Diagn Pathol 2012;16(5):427.
23. Khorana AA, Mangu PB, Berlin J, Engebretson A, Hong TS, Maitra A, et al. Potentially curable pancreatic cancer: American society of clinical oncology clinical practice guideline update. J Clin Oncol 2017;35(20):2324-2328.
24. Callery MP, Chang KJ, Fishman EK, Talamonti MS, William Traverso L, et al. Pretreatment assessment of resectable and borderline resectable pancreatic cancer: expert consensus statement. Ann Surg Oncol 2009;16(7):1727-33.
25. Okusaka T, Nakamura M, Yoshida M, Kitano M, Uesaka K, Ito Y, et al. Clinical practice guidelines for pancreatic cancer 2019 from the Japan pancreas society: A synopsis. Pancreas 2020;49(3):326-335.
26. Committee of the Korean clinical practice guideline for pancreatic cancer and National Cancer Center, Korea. Korean clinical practice guideline for pancreatic cancer 2021: A summary of evidence-based, multi-disciplinary diagnostic and therapeutic approaches. Pancreatology 2021;21(7):1326-1341.
27. Ma C, Yang P, Li J, Bian Y, Wang L, Lu J. Pancreatic adenocarcinoma: variability in measurements of tumor size among computed tomography, magnetic resonance imaging, and pathologic specimens. Abdom Radiol (NY) 2020;45(3):782-788.
28. Bian Y, Jiang H, Cao K, Mms XF, Li J, Ma C, et al. The relationship between microscopic tumor size and CT tumor size in pancreatic ductal adenocarcinoma. Clin Imaging 2021;76:30-37.
29. Arvold ND, Niemierko A, Mamon HJ, Fernandez-del Castillo C, Hong TS. Pancreatic cancer tumor size on CT scan versus pathologic specimen: implications for radiation treatment planning. Int J Radiat Oncol Biol Phys 2011;80(5):1383-90.
30. Hall WA, Mikell JL, Mittal P, Colbert L, Prabhu RS, Kooby DA, et al. Tumor size on abdominal MRI versus pathologic specimen in resected pancreatic adenocarcinoma: implications for radiation treatment planning. Int J Radiat Oncol Biol Phys 2013;86(1):102-7.
31. Bian Y, Jiang H, Zheng J, Shao C, Lu J. Basic pancreatic lesions: Radiologic-pathologic correlation. J Transl Int Med 2022;10(1):18-27.

**Figure legends**

**Fig. 1** Flow chart of the patient selection process.

**Fig. 2** Scatter plots and Bland-Altman plots for tumor size measured by CT and MRI. A: Scatter plot before neoadjuvant therapy (NAT); B: Bland‒Altman plot before NAT; C: Scatter plot after NAT; D: Bland-Altman plot after NAT.

**Fig. 3** Comparisons of the receiver operating characteristic curves for distinguishing the tumor resection margin status (R0, R1) and tumor regression grade (TRG) levels (0-2, 3) with tumor size measured by CT and MRI. A: Distinguishing R0 and R1 preneoadjuvant therapy (NAT), n = 103; B: Distinguishing R0 and R1 after NAT, n = 91; C: Distinguishing TRG levels pre-NAT, n = 103; D: Distinguishing TRG levels after NAT, n = 91.

| **Table 1** Characteristics of 125 patients with pancreatic cancer. | |
| --- | --- |
| Mean age ± SD, years (range) | 60.6 ± 8.5 (33-76) |
| Gender, n (%) |  |
| Male | 72 (57.6%) |
| Female | 53 (42.4%) |
| Location in pancreas, n (%) |  |
| Head/neck | 64 (51.2%) |
| Body/tail | 61 (49.8%) |
| Resectability of pancreatic cancer, n (%) |  |
| Resectable | 55 (44.0%) |
| Borderline resectable | 54 (43.2%) |
| Locally advanced | 16 (12.8%) |
| Histopathologic features of mass, n (%) |  |
| Ductal adenocarcinoma | 114 (91.2%) |
| Adenosquamous carcinoma | 9 (7.2%) |
| Mucinous noncystic carcinoma | 1 (0.8%) |
| Undifferentiated carcinoma | 1 (0.8%) |
| Tumor resection margin status, n (%) |  |
| R0 | 93 (74.4%) |
| R1 | 32 (25.6%) |
| Y of surgery | 2019-2022 |
| N stage, n (%) |  |
| NX | 1 (0.8%) |
| N0 | 63 (50.4%) |
| N1 | 49 (39.2%) |
| N2 | 12 (9.6%) |
| Tumor regression grade, n (%)* |  |
| 0 | 3 (2.4%) |
| 1 | 12 (9.6%) |
| 2 | 73 (58.4%) |
| 3 | 37 (29.6%) |
| Abbreviations: SD: standard deviation  *Grading system of the College of American Pathologists. | |

| **Table 2** Tumor sizes of preneoadjuvant therapy (NAT) and preoperative MRI, CT and pathology. | | | | | |
| --- | --- | --- | --- | --- | --- |
| Parameters | Pre-NAT (n = 103) | | Preoperative (n = 91) | | Pathology  (n = 91) |
|  | MRI | CT | MRI | CT |  |
| Median, mm | 30 | 32 | 22 | 24 | 30 |
| 25th percentile, mm | 22 | 26 | 15 | 15.5 | 20 |
| 75th percentile, mm | 36.5 | 41 | 31 | 32 | 42 |
| Min, mm | 12 | 12 | 0 | 0 | 5 |
| Max, mm | 95 | 94 | 104 | 104 | 100 |

| **Table 3** Comparisons of tumor sizes between MRI and CT before neoadjuvant therapy (NAT) of pancreatic cancer (n=103). | | | | | | | | | | | |
| --- | --- | --- | --- | --- | --- | --- | --- | --- | --- | --- | --- |
| Parameters | NAT methods | | Tumor locations | | TRG levels | | N stages | | Resection margin status | | |
|  | Chemotherapy  (n = 69) | Chemoradiotherapy  (n = 34) | head/neck  (n = 53) | body/tail  (n = 50) | 0-2  (n = 73) | 3  (n = 30) | N0  (n = 51) | N1/N2  (n = 51) | R0  (n = 76) | R1  (n = 27) |  |
| Mean size difference, mm | 1.6 | 1.4 | 1.5 | 1.6 | 1.8 | 0.9 | 2.5 | 0.6 | 2.0 | 0.2 |  |
| Limits of agreement, mm | -10.4 to 13.6 | -8.1 to 11.0 | -9.7 to 12.6 | -9.7 to 12.9 | -7.7 to 11.3 | -13.7 to 15.5 | -7.5 to 12.5 | -11.6 to 12.7 | -9.1 to 13.1 | -11.0 to 11.4 |  |
| ICC | 0.93 | 0.91 | 0.80 | 0.95 | 0.95 | 0.85 | 0.94 | 0.92 | 0.94 | 0.89 |  |
| 95% CI of ICC | 0.89 to 0.96 | 0.82 to 0.95 | 0.68 to 0.88 | 0.91 to 0.97 | 0.92 to 0.97 | 0.71 to 0.93 | 0.90 to 0.97 | 0.86 to 0.95 | 0.90 to 0.96 | 0.78 to 0.95 |  |
| TRG: tumor regression grade; ICC: intraclass correlation coefficient; CI: confidence interval | | | | | | | | | | | |

| **Table 4** Comparisons of tumor sizes between MRI and CT after neoadjuvant therapy (NAT) of pancreatic cancer (n=91). | | | | | | | | | | | |
| --- | --- | --- | --- | --- | --- | --- | --- | --- | --- | --- | --- |
| Parameters | NAT methods | | Tumor locations | | TRG levels | | N stages | | Resection margin status | | |
|  | Chemotherapy  (n = 61) | Chemoradiotherapy  (n = 30) | head/neck  (n = 41) | body/tail  (n = 50) | 0-2  (n = 61) | 3  (n = 30) | N0  (n = 42) | N1/N2  (n = 48) | R0  (n = 69) | R1  (n = 22) |  |
| Mean size difference, mm | 0.9 | 1.0 | 0.4 | 1.4 | 0.6 | 1.5 | 1.4 | 0.5 | 1.6 | -1.4 |  |
| Limits of agreement, mm | -14.2 to 15.9 | -9.1 to 11.1 | -10.9 to 11.6 | -13.9 to 16.6 | -11.8 to 13.0 | -14.3 to 17.3 | -12.0 to 14.7 | -13.4 to 14.5 | -11.3 to 14.6 | -16.0 to 13.3 |  |
| ICC | 0.91 | 0.91 | 0.81 | 0.91 | 0.89 | 0.90 | 0.94 | 0.82 | 0.93 | 0.76 |  |
| 95% CI of ICC | 0.85 to 0.94 | 0.81 to 0.95 | 0.67 to 0.89 | 0.85 to 0.95 | 0.82 to 0.93 | 0.81 to 0.95 | 0.89 to 0.97 | 0.71 to 0.90 | 0.89 to 0.95 | 0.50 to 0.89 |  |
| TRG: tumor regression grade; ICC: intraclass correlation coefficient; CI: confidence interval. | | | | | | | | | | | |

| Table 5 NAT evaluations with the CT and MRI measurements between the groups (PD/SD, CR/PR) based on the RECIST 1.1 guidelines. | | | |
| --- | --- | --- | --- |
|  | *Evaluation of NAT with MRI* | |  |
| *Evaluation of NAT with CT* | PD/SD | CR/PR | Total |
| PD/SD | 26 | 9 | 35(44.9%) |
| CR/PR | 9 | 34 | 43(55.1%) |
| Total | 35(44.9%) | 43(55.1%) | 78 |
| Abbreviations: NAT: neoadjuvant therapy; CR: complete response; PR: partial response; SD: stable disease; PD: progressive disease | | | |

**Clean Manuscript CAIG-D-22-00333R1**

**Tumor Size Measurements of Pancreatic Cancer with Neoadjuvant Therapy Based on RECIST Guidelines: Is MRI as Effective as CT?**

Panpan Yang^1,#^, Kuanzheng Mao^1,2,#^, Yisha Gao^3^, Zhen Wang^1^, Jun Wang^1^, Yufei Chen^4^, Chao Ma^1,4,*^, Yun Bian^1^, Chengwei Shao^1^, Jianping Lu^1,*^

^1^Department of Radiology, Changhai Hospital of Shanghai, Naval Medical University, China; ^2^School of Health Science and Engineering, University of Shanghai for Science and Technology, China; ^3^Department of Pathology, Changhai Hospital of Shanghai, Naval Medical University, China; ^4^College of Electronic and Information Engineering, Tongji University, Shanghai, China

**E-mail of authors:**

Panpan Yang, panwang1112@163.com; Kuanzheng Mao, 541071673@qq.com; Yisha Gao, 241177536@qq.com; Zhen Wang, 314252293@qq.com; Jun Wang, wangjun_ying@sina.com; Yufei Chen, april337@163.com; Chao Ma, mengqihi@gmail.com; Yun Bian, bianyun2012@foxmail.com; Chengwei Shao, chengweishaoch@163.com; Jianping Lu, cjr.lujianping@vip.163.com

**Corresponding to:** Department of Radiology, Changhai Hospital of Shanghai, Naval Medical University, No. 168 Changhai Road, Shanghai 200433, China. cjr.lujianping@vip.163.com (Jianping Lu), mengqihi@gmail.com (Chao Ma)

^#^These authors contributed equally to this work.

**Manuscript Type:** Original research

**Abstract**

**Objectives:** To compare tumor size measurements using CT and MRI in pancreatic cancer (PC) patients with neoadjuvant therapy (NAT).

**Methods:** This study included 125 histologically confirmed PC patients who underwent NAT. The tumor sizes from CT and MRI before and after NAT were compared by using Bland-Altman analyses and intraclass correlation coefficients (ICCs). Variations in tumor size estimates between MRI and CT in relationship to different factors, including NAT methods (chemotherapy, chemoradiotherapy), tumor locations (head/neck, body/tail), tumor regression grade (TRG) levels (0-2, 3), N stages (N0, N1/N2) and tumor resection margin status (R0, R1), were further analysed. The McNemar test was used to compare the efficacy of NAT evaluations based on the CT and MRI measurements according to RECIST 1.1 criteria.

**Results:** There was no significant difference between the median tumor sizes from CT and MRI before and after NAT (P=0.44 and 0.39, respectively). There was excellent agreement in tumor size between MRI and CT, with mean size differences and limits of agreement (LOAs) of 1.5 [-9.6 to 12.7] mm and 0.9 [-12.6 to 14.5] mm before NAT (ICC, 0.93) and after NAT (ICC, 0.91), respectively. For all the investigated factors, there was good or excellent correlation (ICC, 0.76 to 0.95) for tumor sizes between CT and MRI. There was no significant difference in the efficacy evaluation of NAT between CT and MRI measurements (P=1.0).

**Conclusion:** MRI and CT have similar performance in assessing PC tumor size before and after NAT.

**Keywords:** Pancreatic cancer; neoadjuvant therapy, CT; MRI; tumor size

**Introduction**

As a lethal cancer with poor prognosis and high mortality, pancreatic cancer (PC) results in more than 460,000 deaths a year worldwide [1] and may be the second leading cause of death due to cancer by 2030 in the United States [2]. According to the cancer statistics of the United States in 2022, the 5-year survival rate of PC is only 11% [3]. Despite surgical resection is the possible cure method for PC, nowadays the therapeutic principle clearly is not always operation first, considering neoadjuvant therapy (NAT). Most patients with PC have local tumor progression or distant metastasis at diagnosis and miss the opportunity for surgical resection. Only approximately 10%-15% of the patients who are evaluated as having resectable PC can undergo radical surgical resection. More patients are diagnosed with borderline resectable or locally advanced PC [4]. To improve the management of this lethal tumor, on the one hand, earlier diagnosis of resectable PC should be explored, while, on the other hand, new treatment methods should be developed.

NAT is a treatment strategy for resectable, borderline resectable and locally advanced PC that has been emerging considerably [5-9]. The definition of NAT for PC is preoperative chemotherapy with or without radiation therapy [10]. The purpose of NAT for PC is to reduce the tumor stage, improve the rate of R0 resection (a microscopically margin-negative resection), and reduce postoperative recurrence and metastasis [11-14]. Ideal methods for reassessment after NAT for PC are lacking, and currently, the response evaluation criteria in solid tumors (RECIST) are still the main choice for use [9,15]. Investigators could adopt RECIST criteria to assess the treatment outcomes including complete response (CR), partial response (PR), stable disease (SD) and progressive disease (PD) after NAT for PC patients with tumor size measurements. CT and MRI are the commonly used imaging modalities for the diagnosis and evaluation of PC and show similar effects in the staging and diagnosis of pancreatic cancer, both pre-NAT and preoperation without NAT [16]. Different from other solid tumors, the changes in tissue components of PC after NAT, including tumor necrosis, oedema, inflammation, and fibrosis, result in significant effects on CT-image evaluations of tumor treatment and resectability [17]. The accurate staging of tumors with CT or MRI is used not only for the selection of treatment methods but also for accurate tumor measurement to improve outcomes in PC patients after NAT in clinical practice. In this study, we investigated the similarities and differences in tumor measurements between CT and MRI before and after NAT for PC based on RECIST 1.1 criteria.

**Methods**

**Patients**

Our institutional review board approved this retrospective study. We undertook a review of all patients who had undergone pancreatic contrast-enhanced MRI or contrast-enhanced CT and pancreatic tumor resection between April 2019 and December 2021 and identified 1752 pancreatic tumor patients. Among these patients, 125 subjects (72 males, 53 females; mean age: 60.6 ± 8.5 years; range: 33-76 years) had received NAT and had detailed pathological reports. Before NAT, 104 patients underwent both contrast-enhanced CT and contrast-enhanced MRI examinations within an interval of less than 14 days. After NAT, a total of 109 patients underwent both preoperative contrast-enhanced CT and contrast-enhanced MRI examinations, and 17 patients were excluded due to an MRI or CT examination time more than 14 days before the day of the operation. Additionally, a patient with tumor sizes less than 10 mm per pre-NAT CT and MRI measurements was excluded. Finally, this study included 103 and 91 patients who had received both CT and MRI examinations in the pre-NAT group and the finished-NAT group, respectively. Among the 125 enrolled cases, a total of 78 patients who underwent both CT and MRI within an interval of less than 14 days before NAT and subsequently underwent preoperative CT and MRI less than 14 days before the day of the operation were chosen for further analyses (Figure 1, Table 1).

**CT and MRI examinations**

Both the CT and MRI examinations were conducted using pancreatic protocols for all the enrolled patients. For CT examinations, multidetector CT (Toshiba Aquillion One 320, SIEMENS Sensation Cardiac 64, Philips Brilliance iCT 128, United imaging uCT 960+) with a precontrast scan and 3-phase (arterial phase, parenchymal phase, and delayed phase; contrast agent, 90-95 ml with a rate of 2.5-5.5 ml/s) contrast-enhanced scan was utilized, and the axial section thickness was 1.0/1.5 mm. For MRI examinations, 3.0-T MRI systems (Signa HDxt and MR750, GE Healthcare, Milwaukee, USA; Skyra, Siemens, Erlangen, Germany) were used. MRI sequences included coronal and transverse T2-weighted imaging (T2WI), diffusion weighted imaging (DWI) (high b-value 500-1000 s/mm^2^), precontrast T1-weighted imaging (T1WI), and three-phase contrast-enhanced fat-saturated T1-weighted images (contrast agent, 0.1-0.15 mmol/kg with a rate of 2.0-3.0 ml/s).

**Measurement of PC size**

In this study, tumor sizes were measured according to the RECIST 1.1 criteria (15). The maximum dimension tumor size described in the radiology reports (CT and MRI) was regarded as the tumor size of the PC. To investigate whether there was difference between the tumor size on radiology reports and the re-measurements, a radiologist who was unaware of the results of pathology reports and radiology reports repeated the measurements of PC sizes both on CT and MRI before and after NAT with 103 patients and 91 patients, respectively. With an interval of six weeks, the radiologist repeated the measurements of PC sizes to evaluate the intraobserver agreement. Evaluation of the target lesions of PC based on pre-NAT CT/MRI and preoperative CT/MRI was performed by the RECIST 1.1 guidelines, and the PC patients were divided into four groups including CR, PR, SD and PD after NAT.

**Pathologic response to NAT**

The structured pathological reports for NAT of PC were used in our hospital. Each report recorded the following details: general description, details of materials taken, morphological description, margins and neighbours, lymph node metastasis, diagnosis, tumor regression grade (TRG), etc. The TRG of PC after NAT was performed by the grading system of the College of American Pathologists (CAP), which divides TRG into 4 levels (Grading 0 to 3) according to the ratio of residual tumor cells and the stroma [18]. Grade 0 indicates complete response after NAT of PC and no surviving tumor cells, and grade 3 indicates the NAT was ineffective and many tumor cells remained. The definitions of R0 and R1 were determined according to whether there was absence of tumor cell infiltration within 1 mm of the resection margin. Two pathologists analysed pathological images to issue pathological reports.

**Statistical analysis**

Statistical analyses were performed by MedCalc version 13.0.0.0 (MedCalc Software, Ostend, Belgium). The reproducibility between the tumor size on radiology reports and re-measurements and intraobserver agreement of the measurements of PC size were evaluated by using Bland-Altman analyses (19) and intraclass correlation coefficients (ICCs: 0-0.20 = poor correlation, etc.) (20). Before and after NAT, the differences in the PC tumor sizes between MRI and CT were analysed using the Wilcoxon test (paired samples) with MRI size corrections based on a 5% noninferiority margin. The variability in tumor size measurements of PC on MRI and CT were analyzed by Bland-Altman analyses and ICCs. The differences and variability in tumor sizes between MRI and CT in relationship to different factors, including NAT methods (chemotherapy, chemoradiotherapy), tumor locations (head/neck, body/tail), TRG levels (0-2, 3), N stages (N0, N1/N2) and tumor resection margin status (R0, R1), were further analysed. A receiver operating characteristic (ROC) curve was used to investigate differences in tumor size as measured by CT and MRI to differentiate the tumor resection margin status (R0, R1) and TRG levels (0-2, 3). The discrepancy in NAT evaluations based on the CT and MRI measurements between the groups (PD/SD, CR/PR) was also analysed with the McNemar test (paired samples). The statistical significance levels were set at a P value < 0.05.

**Results**

**Patients**

The characteristics of the 125 patients are detailed in Table 1. There were 72 men and 53 women with a mean age of 60.6 ± 8.5 (33-76), and 51.2% (64 of 125 patients) of the tumors were located in the pancreatic head/neck. Except for one case without peripancreatic lymphadenectomy, metastases to regional lymph nodes were found in 50% (62 of 124 patients) of patients on histological examinations. Among the cases enrolled in this study, there were 55, 54 and 16 patients with resectable, borderline resectable and locally advanced PC, respectively.

**Tumor size measurements between CT and MRI**

There were excellent agreements in the PC sizes on radiology reports and re-measurements, with mean size differences and LOAs of -1.5 [-10.4 to 7.4] mm and -0.9 [-12.5 to 10.6] mm for both CT and MRI before NAT (ICCs, 0.95 and 0.92) and -2.1 [-13.9 to 9.7] mm and -2.2 [-17.2 to 12.9] mm after NAT (ICCs, 0.92 and 0.87), respectively. For the twice measurements of PC size by a radiology, there were excellent agreements with mean size differences and LOAs of -0.3 [-6.8 to 6.1] mm and -0.2 [-7.2 to 6.7] mm for both CT and MRI before NAT (ICCs, 0.98 and 0.97) and -0.5 [-7.0 to 6.0] mm and -0.2 [-7.2 to 6.8] mm after NAT (ICCs, 0.98 and 0.97), respectively.

There was no significant difference in the median tumor sizes on CT and MRI before and after NAT with 103 (P = 0.44) and 91 (P = 0.39) subjects, respectively. There were excellent agreements in the tumor sizes on MRI and CT, with mean size differences and LOAs of 1.5 [-9.6 to 12.7] mm and 0.9 [-12.6 to 14.5] mm before NAT (ICC, 0.93) and after NAT (ICC, 0.91), respectively (Figure 2). It is worth noting that the median tumor sizes based on preoperative MRI and CT were 22.0 mm and 24.0 mm, respectively, and both the mean CT and MRI sizes of PC were significantly smaller than the pathological size (median, 30.0 mm) (both P < 0.001) (Table 2).

For the tumor size measurements of PC on both the pre-NAT and preoperative CT and MRI images given different factors, including NAT methods (chemotherapy, chemoradiotherapy), tumor locations (head/neck, body/tail), TRG levels (0-2, 3), N stages (N0, N1/N2) and tumor resection margin status (R0, R1), there were good or excellent correlations under all the factors, with ICCs ranging from 0.76 to 0.95, the mean size differences ranging from -1.4 mm to 2.5 mm and a range of LOAs for tumor size measurements < ± 16 mm (Tables 3 and 4); that is, there was good correlation and consistency between CT and MRI tumor sizes in the subgroup analysis mentioned above.

**Tumor resection margin status and TRG levels in size estimates**

The area under the curve (AUC) was not significantly different for distinguishing between the R0 and R1 groups using tumor sizes with pre-NAT (AUC, 0.57 and 0.58; P = 0.64) or preoperative CT and MRI measurements (AUC, 0.60 and 0.66; P = 0.08). Additionally, for the comparisons of AUC of the TRG levels, no significant differences were observed between the two groups of TRG with pre-NAT (AUC, 0.57 and 0.54; P = 0.42) or preoperative CT and MRI measurements (AUC, 0.74 and 0.72; P = 0.48) (Figure 3).

**Efficacy evaluation of NAT with CT and MRI measurements**

Based on tumor sizes from the CT and MRI radiology reports, 77% (60/78) of patients had the same efficacy evaluation group with the guideline of RECIST 1.1. McNemar test (paired samples) results showed no statistically significant difference for the efficacy evaluation of NAT with CT and MRI measurements (P = 1.0) (Table 5). With the first measurements of PC sizes of the radiologist, there was about 85% (66/78) of the patients had the same response category group. No significant difference was also found in the efficacy evaluation of NAT between CT and MRI measurements (P = 0.15).

**Discussion**

NAT has been a considerable and important treatment strategy for PC patients. It reduces the tumor stage, allows patients with borderline resectable and locally advanced PC to obtain surgical opportunities and increases the rate of negative margins (R0 resection) with NAT [11-14]. After NAT, PC cell damage and NAT-induced tumor interstitial fibrosis are similar to the fibroproliferative matrix of PC itself and pancreatitis fibrosis. Traditional imaging, such as CT and MRI, cannot accurately evaluate the efficacy of NAT because it cannot distinguish between fibrous tissue and cancer tissue after NAT [21,22]. In addition, it is still difficult to assess whether the tumor shows regression. Nevertheless, both CT and MRI are still the most important tools for the diagnosis and evaluation of PC with treatment [9]. Many international guidelines recommend using CT first in the diagnosis of suspected PC [5,23-26]. MRI generally shows the same ability of PC detection as CT and may have a potential to reveal structural nature of pancreas more precisely than CT in the patients whose lesions are hard to be recognized in traditional CT.. The RECIST 1.1 method is widely adopted to evaluate the efficacy of solid tumor therapy by measuring the change in tumor size based on CT or MRI before and after treatment. Therefore, it is important to assess PC size with NAT and select the most effective treatment in clinical practice. The importance of tumor size measurement after NAT for PC has been emphasized in the AJR Expert Narrative Panel Review [9]. In this study, our results showed that MRI and CT have similar performance in assessing PC tumor size before and after NAT.

To further confirm our findings, we performed tumor size analyses between CT and MRI measurements in PC patients with NAT by five stratifications, including NAT methods (chemotherapy, chemoradiotherapy), tumor locations (head/neck, body/tail), TRG levels (0-2, 3), N stages (N0, N1/N2) and tumor resection margin status (R0, R1). The results were similar to the conclusions from assessment of the overall cases before and after NAT. In the current study, the average interval between CT and MRI examinations performed preoperatively after NAT was 1.6 days, and the interval between preoperative CT or MRI examinations and the day of operations was 5 days. It is worth noting that the preoperative CT and MRI tumor sizes after NAT were significantly smaller than the pathological size of PC (both P < 0.001), with a median tumor size underestimation of approximately 8 mm and 6 mm for MRI and CT, respectively. These findings are similar to our previous studies [27,28] and those of Arnold et al [29] and Hall et al [30], in which resectable PC without NAT had CT or MRI sizes smaller than the pathological sizes. Possible reasons for the tumor CT and MRI sizes of resectable PC being inconsistent with the pathological conclusions have been detailed in our previous reports [27,28]. In this study, PC was highly dispersed and presented a leap-forward growth pattern. Tumor cells are often distributed in a wide range of stroma, especially around the tumor. In pathological examination, it is usually impossible to distinguish between NAT-induced fibrosis and dense fibrous stroma, so it is difficult to define the tumor contour before and after NAT, and imaging evaluation often underestimates the number of tumor cells scattered around the tumor.

The tumor resection margin status and TRG levels of PC are important clinical indicators for NAT. In the current study, there were no significant differences in distinguishing between the R0 and R1 groups or TRG levels using tumor sizes from pre-NAT or preoperative CT and MRI measurements. Additionally, there was no significant difference in the efficacy evaluation of NAT between CT and MRI measurements, and the measurements of PC sizes by the same radiology before and after NAT will improve the accuracy of response category between CT and MRI. These results further confirm that MRI and CT have similar performance in assessing PC tumor size after NAT.

Tumor size and tumor stage after NAT are key indicators for evaluating efficacy. Our study has compared the difference between the imaging and pathological sizes of tumors after NAT for PC. In pathological analysis after NAT for PC, the specification of pathological sampling has not been unified. In this study, the pathological diagnosis of 3 cases was complete regression after NAT, but nodular-like mass could still be observed in gross, and there were also corresponding imaging findings on CT and MRI. Traditional pathological small sections have errors in measuring the size of PC after NAT, and whole-mount pathological analyses will have better application prospects in T staging [31]. Multidisciplinary clinics are very important for NAT for PC. It is expected that there will be standardized methods developed from imaging examination, follow-up during treatment and pathology analysis after NAT to provide more accurate strategies for treatment of PC patients and to improve treatment effects.

Our study had some limitations. First, this study was designed as a retrospective study; the aspects that we could control were limited. Second, the neoadjuvant methods used in this study were not uniform. For example, FOLFIRINOX, mFOLFIRINOX, albumin-bound paclitaxel plus gemcitabine were used in chemotherapy. Due to sample size limitations, we did not further stratify different chemotherapy regimens.

**Conclusion**

Both CT and MRI have significant advantages in the assessment of PC. Our findings showed that MRI and CT have similar efficacy in the assessment of tumor size before and after NAT for PC, and MRI may have more advantages in multiple follow-ups because of the absence of ionizing radiation. Therefore, the guidelines and application value of PC size assessment after NAT need to be further clarified and studied to make better treatment decisions in clinical practice.

**Abbreviations**

CT: computed tomography; MRI: magnetic resonance imaging; PC: pancreatic cancer; NAT: neoadjuvant therapy; ICC: intraclass correlation coefficient; TRG: tumor regression grade; RECIST: response evaluation criteria in solid tumors; LOA: limits of agreement; CR: complete response, PR: partial response, SD: stable disease; PD: progressive disease; ROC: receiver operating characteristic; AUC: area under the curve

**Acknowledgements**

We thank Mr. Wang Lizhi for his help in data statistics and analysis.

**Authors’ contributions**

Yang P, Mao K, Gao Y, Wang Z, Wang J, Ma C, and Bian Y performed the majority of experiments, made substantial contributions to the data analysis and interpretation, and wrote the manuscript draft; Ma C, Chen Y, Shao C and Lu J participated in the design of the study and made substantial contribution to data analysis; Ma C and Lu J made substantial contributions to the study conception and design, critically revised the manuscript draft for important intellectual content, and gave final approval of the version to be published; all the authors read and approved the final manuscript.

**Funding**

This work was supported by the National Natural Science Foundation of China [82171915, 82171930, 62173252]; 234 Platform Discipline Consolidation Foundation Project of Changhai Hospital [2020YPT001]; Shanghai Science and Technology Innovation Action Plan Medical Innovation Research Project [20Y11912500].

**Data availability**

The research dataset for the current study is available from the corresponding author upon reasonable request.

**Declarations
Ethics approval and consent to participate**

This study was approved by our Institutional Review Board (Shanghai Changhai Hosptial Ethics committee). Signed written informed consent was waived from all participants.

**Consent for publication**

Not applicable.

**Competing interests**

The authors declare that they have no competing interests.

**References**

32. Sung H, Ferlay J, Siegel RL, Laversanne M, Soerjomataram I, Jemal A, et al. Global Cancer Statistics 2020: GLOBOCAN estimates of incidence and mortality worldwide for 36 cancers in 185 Countries. CA Cancer J Clin 2021;71(3):209-249.
33. Saad AM, Turk T, Al-Husseini MJ, Abdel-Rahman O. Trends in pancreatic adenocarcinoma incidence and mortality in the United States in the last four decades; a SEER-based study. BMC Cancer 2018;18(1):688.
34. Siegel RL, Miller KD, Fuchs HE, Jemal A. Cancer statistics, 2022. CA Cancer J Clin 2022;72(1):7-33.
35. Park W, Chawla A, O'Reilly EM. Pancreatic Cancer: A Review. JAMA 2021;326(9):851-862.
36. Tempero MA, Malafa MP, Al-Hawary M, Behrman SW, Benson AB, Cardin DB, et al. Pancreatic adenocarcinoma, Version 2.2021, NCCN clinical practice guidelines in oncology. J Natl Compr Canc Netw 2021;19(4):439-457.
37. Kulkarni NM, Soloff EV, Tolat PP, Sangster GP, Fleming JB, Brook OR, et al. White paper on pancreatic ductal adenocarcinoma from society of abdominal radiology's disease-focused panel for pancreatic ductal adenocarcinoma: Part I, AJCC staging system, NCCN guidelines, and borderline resectable disease. Abdom Radiol (NY) 2020;45(3):716-728.
38. Gugenheim J, Crovetto A, Petrucciani N. Neoadjuvant therapy for pancreatic cancer. Updates Surg 2022;74(1):35-42.
39. Versteijne E, van Dam JL, Suker M, Janssen QP, Groothuis K, Akkermans-Vogelaar JM, et al. Neoadjuvant chemoradiotherapy versus upfront surgery for resectable and borderline resectable pancreatic cancer: Long-term results of the dutch randomized PREOPANC trial. J Clin Oncol 2022;40(11):1220-1230.
40. Soloff EV, Al-Hawary MM, Desser TS, Fishman EK, Minter RM, Zins M. Imaging assessment of pancreatic cancer resectability after neoadjuvant therapy: *AJR* Expert Panel Narrative Review. AJR Am J Roentgenol 2022;218(4):570-581.
41. Heinrich S, Lang H. Neoadjuvant therapy of pancreatic cancer: Definitions and benefits. Int J Mol Sci 2017;18(8):1622.
42. Reni M, Balzano G, Zanon S, Zerbi A, Rimassa L, Castoldi R, et al. Safety and efficacy of preoperative or postoperative chemotherapy for resectable pancreatic adenocarcinoma (PACT-15): a randomised, open-label, phase 2-3 trial. Lancet Gastroenterol Hepatol 2018;3(6):413-423.
43. Townend P, de Reuver PR, Chua TC, Mittal A, Clark SJ, Pavlakis N, et al. Histopathological tumor viability after neoadjuvant chemotherapy influences survival in resected pancreatic cancer: analysis of early outcome data. ANZ J Surg 2018;88(3):E167-E172.
44. Versteijne E, Vogel JA, Besselink MG, Busch ORC, Wilmink JW, Daams JG, et al. Meta-analysis comparing upfront surgery with neoadjuvant treatment in patients with resectable or borderline resectable pancreatic cancer. Br J Surg 2018;105(8):946-958.
45. Versteijne E, Suker M, Groothuis K, Akkermans-Vogelaar JM, Besselink MG, Bonsing BA, et al. Preoperative chemoradiotherapy versus immediate surgery for resectable and borderline resectable pancreatic cancer: results of the dutch randomized phase III PREOPANC trial. J Clin Oncol 2020;38(16):1763-1773.
46. Schwartz LH, Litière S, de Vries E, Ford R, Gwyther S, Mandrekar S, et al. RECIST 1.1-Update and clarification: From the RECIST committee. Eur J Cancer 2016;62:132-7.
47. Treadwell JR, Zafar HM, Mitchell MD, Tipton K, Teitelbaum U, Jue J. Imaging tests for the diagnosis and staging of pancreatic adenocarcinoma: A meta-analysis. Pancreas 2016;45(6):789-95.
48. White RR, Paulson EK, Freed KS, Keogan MT, Hurwitz HI, Lee C, et al. Staging of pancreatic cancer before and after neoadjuvant chemoradiation. J Gastrointest Surg 2001;5(6):626-33.
49. Washington MK, Berlin J, Branton P, Burgart LJ, Carter DK, Fitzgibbons PL, et al. Protocol for the examination of specimens from patients with primary carcinoma of the colon and rectum. Arch Pathol Lab Med 2009;133(10):1539-51.
50. Bland JM, Altman DG. Statistical methods for assessing agreement between two methods of clinical measurement. Lancet 1986; 1(8476):307-10.
51. Cohen J. Weighted kappa: nominal scale agreement with provision for scaled disagreement or partial credit. Psychol Bull 1968;70(4):213-20.
52. Verbeke CS, Knapp J, Gladhaug IP. Tumor growth is more dispersed in pancreatic head cancers than in rectal cancer: implications for resection margin assessment. Histopathology 2011;59(6):1111-21.
53. Wang H, Zhao Q. Pathologic complete response to neoadjuvant therapy in patients with pancreatic ductal adenocarcinoma is associated with better prognosis. Ann Diagn Pathol 2012;16(5):427.
54. Khorana AA, Mangu PB, Berlin J, Engebretson A, Hong TS, Maitra A, et al. Potentially curable pancreatic cancer: American society of clinical oncology clinical practice guideline update. J Clin Oncol 2017;35(20):2324-2328.
55. Callery MP, Chang KJ, Fishman EK, Talamonti MS, William Traverso L, et al. Pretreatment assessment of resectable and borderline resectable pancreatic cancer: expert consensus statement. Ann Surg Oncol 2009;16(7):1727-33.
56. Okusaka T, Nakamura M, Yoshida M, Kitano M, Uesaka K, Ito Y, et al. Clinical practice guidelines for pancreatic cancer 2019 from the Japan pancreas society: A synopsis. Pancreas 2020;49(3):326-335.
57. Committee of the Korean clinical practice guideline for pancreatic cancer and National Cancer Center, Korea. Korean clinical practice guideline for pancreatic cancer 2021: A summary of evidence-based, multi-disciplinary diagnostic and therapeutic approaches. Pancreatology 2021;21(7):1326-1341.
58. Ma C, Yang P, Li J, Bian Y, Wang L, Lu J. Pancreatic adenocarcinoma: variability in measurements of tumor size among computed tomography, magnetic resonance imaging, and pathologic specimens. Abdom Radiol (NY) 2020;45(3):782-788.
59. Bian Y, Jiang H, Cao K, Mms XF, Li J, Ma C, et al. The relationship between microscopic tumor size and CT tumor size in pancreatic ductal adenocarcinoma. Clin Imaging 2021;76:30-37.
60. Arvold ND, Niemierko A, Mamon HJ, Fernandez-del Castillo C, Hong TS. Pancreatic cancer tumor size on CT scan versus pathologic specimen: implications for radiation treatment planning. Int J Radiat Oncol Biol Phys 2011;80(5):1383-90.
61. Hall WA, Mikell JL, Mittal P, Colbert L, Prabhu RS, Kooby DA, et al. Tumor size on abdominal MRI versus pathologic specimen in resected pancreatic adenocarcinoma: implications for radiation treatment planning. Int J Radiat Oncol Biol Phys 2013;86(1):102-7.
62. Bian Y, Jiang H, Zheng J, Shao C, Lu J. Basic pancreatic lesions: Radiologic-pathologic correlation. J Transl Int Med 2022;10(1):18-27.

**Figure legends**

**Fig. 1** Flow chart of the patient selection process.

**Fig. 2** Scatter plots and Bland-Altman plots for tumor size measured by CT and MRI. A: Scatter plot before neoadjuvant therapy (NAT); B: Bland‒Altman plot before NAT; C: Scatter plot after NAT; D: Bland-Altman plot after NAT.

**Fig. 3** Comparisons of the receiver operating characteristic curves for distinguishing the tumor resection margin status (R0, R1) and tumor regression grade (TRG) levels (0-2, 3) with tumor size measured by CT and MRI. A: Distinguishing R0 and R1 preneoadjuvant therapy (NAT), n = 103; B: Distinguishing R0 and R1 after NAT, n = 91; C: Distinguishing TRG levels pre-NAT, n = 103; D: Distinguishing TRG levels after NAT, n = 91.

| **Table 1** Characteristics of 125 patients with pancreatic cancer. | |
| --- | --- |
| Mean age ± SD, years (range) | 60.6 ± 8.5 (33-76) |
| Gender, n (%) |  |
| Male | 72 (57.6%) |
| Female | 53 (42.4%) |
| Location in pancreas, n (%) |  |
| Head/neck | 64 (51.2%) |
| Body/tail | 61 (49.8%) |
| Resectability of pancreatic cancer, n (%) |  |
| Resectable | 55 (44.0%) |
| Borderline resectable | 54 (43.2%) |
| Locally advanced | 16 (12.8%) |
| Histopathologic features of mass, n (%) |  |
| Ductal adenocarcinoma | 114 (91.2%) |
| Adenosquamous carcinoma | 9 (7.2%) |
| Mucinous noncystic carcinoma | 1 (0.8%) |
| Undifferentiated carcinoma | 1 (0.8%) |
| Tumor resection margin status, n (%) |  |
| R0 | 93 (74.4%) |
| R1 | 32 (25.6%) |
| Y of surgery | 2019-2022 |
| N stage, n (%) |  |
| NX | 1 (0.8%) |
| N0 | 63 (50.4%) |
| N1 | 49 (39.2%) |
| N2 | 12 (9.6%) |
| Tumor regression grade, n (%)* |  |
| 0 | 3 (2.4%) |
| 1 | 12 (9.6%) |
| 2 | 73 (58.4%) |
| 3 | 37 (29.6%) |
| Abbreviations: SD: standard deviation  *Grading system of the College of American Pathologists. | |

| **Table 2** Tumor sizes of preneoadjuvant therapy (NAT) and preoperative MRI, CT and pathology. | | | | | |
| --- | --- | --- | --- | --- | --- |
| Parameters | Pre-NAT (n = 103) | | Preoperative (n = 91) | | Pathology  (n = 91) |
|  | MRI | CT | MRI | CT |  |
| Median, mm | 30 | 32 | 22 | 24 | 30 |
| 25th percentile, mm | 22 | 26 | 15 | 15.5 | 20 |
| 75th percentile, mm | 36.5 | 41 | 31 | 32 | 42 |
| Min, mm | 12 | 12 | 0 | 0 | 5 |
| Max, mm | 95 | 94 | 104 | 104 | 100 |

| **Table 3** Comparisons of tumor sizes between MRI and CT before neoadjuvant therapy (NAT) of pancreatic cancer (n=103). | | | | | | | | | | | |
| --- | --- | --- | --- | --- | --- | --- | --- | --- | --- | --- | --- |
| Parameters | NAT methods | | Tumor locations | | TRG levels | | N stages | | Resection margin status | | |
|  | Chemotherapy  (n = 69) | Chemoradiotherapy  (n = 34) | head/neck  (n = 53) | body/tail  (n = 50) | 0-2  (n = 73) | 3  (n = 30) | N0  (n = 51) | N1/N2  (n = 51) | R0  (n = 76) | R1  (n = 27) |  |
| Mean size difference, mm | 1.6 | 1.4 | 1.5 | 1.6 | 1.8 | 0.9 | 2.5 | 0.6 | 2.0 | 0.2 |  |
| Limits of agreement, mm | -10.4 to 13.6 | -8.1 to 11.0 | -9.7 to 12.6 | -9.7 to 12.9 | -7.7 to 11.3 | -13.7 to 15.5 | -7.5 to 12.5 | -11.6 to 12.7 | -9.1 to 13.1 | -11.0 to 11.4 |  |
| ICC | 0.93 | 0.91 | 0.80 | 0.95 | 0.95 | 0.85 | 0.94 | 0.92 | 0.94 | 0.89 |  |
| 95% CI of ICC | 0.89 to 0.96 | 0.82 to 0.95 | 0.68 to 0.88 | 0.91 to 0.97 | 0.92 to 0.97 | 0.71 to 0.93 | 0.90 to 0.97 | 0.86 to 0.95 | 0.90 to 0.96 | 0.78 to 0.95 |  |
| TRG: tumor regression grade; ICC: intraclass correlation coefficient; CI: confidence interval | | | | | | | | | | | |

| **Table 4** Comparisons of tumor sizes between MRI and CT after neoadjuvant therapy (NAT) of pancreatic cancer (n=91). | | | | | | | | | | | |
| --- | --- | --- | --- | --- | --- | --- | --- | --- | --- | --- | --- |
| Parameters | NAT methods | | Tumor locations | | TRG levels | | N stages | | Resection margin status | | |
|  | Chemotherapy  (n = 61) | Chemoradiotherapy  (n = 30) | head/neck  (n = 41) | body/tail  (n = 50) | 0-2  (n = 61) | 3  (n = 30) | N0  (n = 42) | N1/N2  (n = 48) | R0  (n = 69) | R1  (n = 22) |  |
| Mean size difference, mm | 0.9 | 1.0 | 0.4 | 1.4 | 0.6 | 1.5 | 1.4 | 0.5 | 1.6 | -1.4 |  |
| Limits of agreement, mm | -14.2 to 15.9 | -9.1 to 11.1 | -10.9 to 11.6 | -13.9 to 16.6 | -11.8 to 13.0 | -14.3 to 17.3 | -12.0 to 14.7 | -13.4 to 14.5 | -11.3 to 14.6 | -16.0 to 13.3 |  |
| ICC | 0.91 | 0.91 | 0.81 | 0.91 | 0.89 | 0.90 | 0.94 | 0.82 | 0.93 | 0.76 |  |
| 95% CI of ICC | 0.85 to 0.94 | 0.81 to 0.95 | 0.67 to 0.89 | 0.85 to 0.95 | 0.82 to 0.93 | 0.81 to 0.95 | 0.89 to 0.97 | 0.71 to 0.90 | 0.89 to 0.95 | 0.50 to 0.89 |  |
| TRG: tumor regression grade; ICC: intraclass correlation coefficient; CI: confidence interval. | | | | | | | | | | | |

| Table 5 NAT evaluations with the CT and MRI measurements between the groups (PD/SD, CR/PR) based on the RECIST 1.1 guidelines. | | | |
| --- | --- | --- | --- |
|  | *Evaluation of NAT with MRI* | |  |
| *Evaluation of NAT with CT* | PD/SD | CR/PR | Total |
| PD/SD | 26 | 9 | 35(44.9%) |
| CR/PR | 9 | 34 | 43(55.1%) |
| Total | 35(44.9%) | 43(55.1%) | 78 |
| Abbreviations: NAT: neoadjuvant therapy; CR: complete response; PR: partial response; SD: stable disease; PD: progressive disease | | | |
